# A 3D Printed Hanging Drop Dripper for Tumor Spheroids Analysis Without Recovery

**DOI:** 10.1038/s41598-019-56241-0

**Published:** 2019-12-23

**Authors:** Liang Zhao, Jidong Xiu, Yang Liu, Tianye Zhang, Wenjie Pan, Xiaonan Zheng, Xueji Zhang

**Affiliations:** 1 Institute of Precision Medicine and Health, University of Science and Technology Beijing, Beijing, 100083 China; 2 Research Center for Bioengineering and Sensing Technology, University of Science and Technology Beijing, Beijing, 100083 China; 3 Beijing Key Laboratory for Bioengineering and Sensing Technology University of Science and Technology Beijing, Beijing, 100083 China; 4 School of Chemistry and Biological Engineering, University of Science and Technology Beijing, Beijing, 100083 China

**Keywords:** Lab-on-a-chip, Assay systems

## Abstract

Compared with traditional monolayer cell culture, the three-dimensional tumor spheroid has emerged as an essential *in vitro* model for cancer research due to the recapitulation of the architecture and physiology of solid human tumors. Herein, by implementing the rapid prototyping of a benchtop 3D printer, we developed a new strategy to generate and analyze tumor spheroids on a commonly used multi-well plate. In this method, the printed artifact can be directly mounted on a 96/384-well plate, enables hanging drop-based spheroid formation, avoiding the tedious fabrication process from micromechanical systems. Besides long-term spheroid culture (20 days), this method supports subsequent analysis of tumor spheroid by seamlessly dripping from the printed array, thereby eliminating the need for spheroids retrieval for downstream characterization. We demonstrated several tumor spheroid-based assays, including tumoroid drug testing, metastasis on or inside extracellular matrix gel, and tumor transendothelial (TEM) assay. Based on quantitative phenotypical and molecular analysis without any precarious retrieval and transfer, we found that the malignant breast cancer (MDA-MB-231) cell aggregate presents a more metastatic morphological phenotype than the non-malignant breast cancer (MCF-7) and colonial cancer (HCT-116) cell spheroid, and shows an up-regulation of epithelial-mesenchymal transition (EMT) relevant genes (fold change > 2). Finally, we validated this tumor malignancy by the TEM assay, which could be easily performed using our approach. This methodology could provide a useful workflow for expediting tumoroid modeled *in vitro* assay, allowing the “Lab-on-a-Cloud” scenario for routine study.

## Introduction

In the cellular biological study, it is generally recognized that the flask-based cell culture approaches are intrinsically not able to recreate cellular architectures found in organisms^1–3^. Currently, however, most initial screening in drug development is still using the 2D-model-based cell assay, which compromises its clinical relevance and hampers predictive capability for efficacy^4,5^. 3D cell cultures are considered as an effective way to address the insufficient recapitulation of the monolayer cell cultures. Concisely, cell culture in 3D mode could be categorized into two realms: 3D cell culture within the fabricated scaffold, and scaffold-free cell aggregates culture^6^. Although the scaffold-materials-based approach allows remolding a 3D environment by supporting cells with synthetic hydrogel or natural extracellular matrices^7,8^, it is limited in recapitulating *in vivo* cytoarchitecture and organization, because cells in the scaffold are challenging to reach a high cellular-density, such as tumors^9^.

The cellular spheroid formation is one of the most straightforward methods to recreate *in vivo* like cell culture-based assay for therapeutically orientated biomedical study^10^. Conventional approaches to produce cell aggregates, including culturing cell in stirring suspension^11^, round bottom non-adherent plate^12^, by magnetic levitation^13^, and hanging drop^14^, are hampered by the limitations like the variation in spheroids size, cell number, labor-intensive, high-shear force, and difficulties on massive production^15^. Recently, some microfabrication based methods, such as microwell^16–18^, microfluidics^19,20^, and microfabricated hanging drop^21–23^, have gained lots of attention due to the formation of a large amount of well-controlled aggregates with uniform size, less laborious, and amenable to high throughput screening^24^. However, to produce such platforms, expensive and time-consuming photolithography or micro-molding fabrication is still an indispensable requisite in those methodologies, and thus are closed-source technologies and not a cost-effective way to perform a micro tissue-based assay.

Herein, we developed a desktop 3D-printed hanging-drop dripper (3D-phd) device that allows riding on 96/384-well plate for uniformly generating cell spheroids, long-term culturing, drug testing, and *in situ* analysis of tumor migration and invasion in ECM niche. Our approach advances frequently used the hanging drop method towards an open-source and flexible method that can be easily manufactured by a standard benchtop 3D printer. The concept of printing out biological assay used device and combining with standard culture plate offers following advantages: (i) enhanced reproducibility and robustness by harnessing additive manufacture workflow; (ii) ultrafast and simple producing device with on-cloud STL format file; (iii) high flexibility allows quick design change of prototype within hours; (iv) facile downstream analysis due to adapting of standard tool such as 96/384-well plate; (v) more modules could be further added by 3D printing to fulfill integration of heterogeneous culturing of different spheroids or so-called “body-on-a-chip”^25^ could be reformed as “body-on-cloud”. In addition, due to the dripping-like collection of cultured spheroids, our platform is seamlessly compatible with many assays, such as drug-induced cell death by inverted confocal microscopy, metastasis on ECM surface or embedded in ECM gel, and tumor cell transendothelial migration within ECM microenvironment. To our knowledge, this is the first demonstration of a 3D printed device for hanging drop generating cell aggregates and subsequently used for a variety of tumor-based assays without recovery.

## Results

### Strategy of 3D-phd

**Figure 1:**
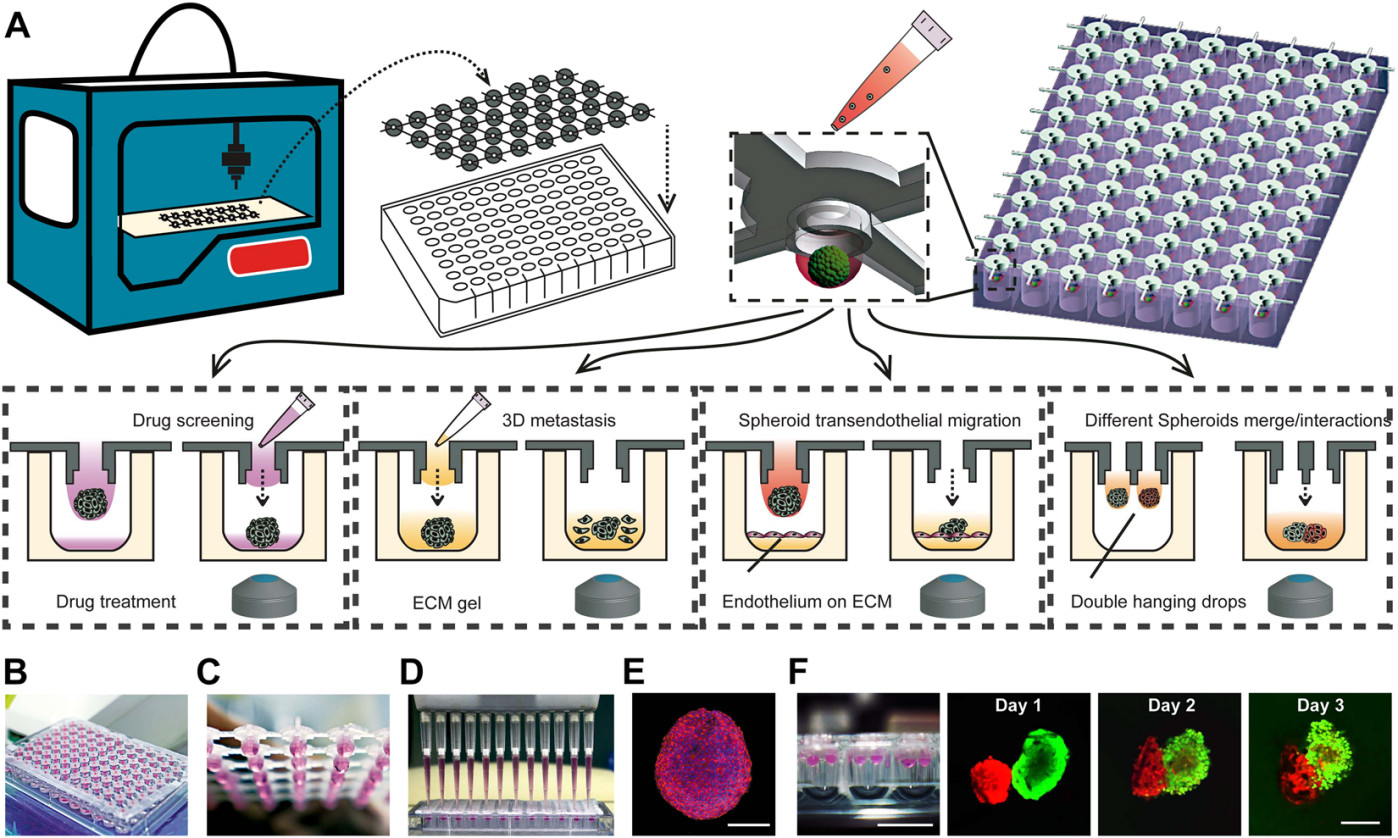
3D-printed hanging drop dripper (3D-phd) for tumor spheroids study. (**A**) The workflow of 3D-phd for studying tumor spheroid generation, drug-induced cell death, and metastasis in extracellular matrix gel. The device has been printed and directly used for cell spheroid generation on a 96/384 well culture plate. Closeup artwork shows a detailed structure of an individual spheroid culture site with a cell aggregate. Four different assays have been developed based on 3D-phd platform: (i) Specific components for drug screening can be added through device, and cell toxicity imaging was directly acquired by dripping down treated cell spheroid with specific staining; (ii) 3D metastasis of cell spheroid on/in matrix could be performed on a matrix gel-coated plate or dropping off with cell-matrix gel solution directly; (iii) The 3D tumor spheroid based transendothelial migration analysis; (iv) Heterotypical spheroids interaction by using the double nozzles 3D-phd. Photograph of 3D-phd mounted on a 96 well plate shown in (**B**), and the closeup of the hanging drops on the device has been shown in (**C**). The standard pipetting operation for medium changing or dropping down is displayed in (**D**). (**E**) A 3D-phd cultured MCF-7 cell spheroid was stained by phalloidin-rhodamine (red) and Hoechst-33342 (blue). The staining was performed after 24 h culture. Scale bar is 200 µm. (**F**) The photograph of the hanging drops on double nozzles device (left) mounted on a 96-well U-shape low attachment plate. The time-lapse fluorescence images of the fusion dynamics of two different spheroids. The spheroids have been directly dropped down into the well, MCF-7 (CellTracker Red labeled) and MDA-MB231 (CellTracker Green labeled) tumor, respectively. Scale bar is 10 mm in the left photograph and 200 μm in fluorescence micrograph.

On our 3D-phd array, each cell spheroid culture site (SCS) was designed to align with the projective center of each culture well to facilitate standard liquid handling and following operation, including medium changing and pipette dripping down to the bottom (Fig. 1A). In Figs. S1–3, the two-dimensional orthographic view shows the design details for single SCS architecture. To prevent evaporation, we added some of the culture medium into the bottom well. To better illustrate the device configuration, we showed both the animation of the whole 3D-phd (Fig. 1A) and real pictures of the array with the hanging drops (Fig. 1B–D, F, Figs. S4–6). After mounted on a 24/96/384 well culture plate, cell suspension with adjusted density was pipetted into each SCS on a 3D-phd device, and the self-organized spherical cell cluster will be generated within 12–24 h (Fig. S7). Figure 1A illustrated this strategy of using this platform to execute the *in situ* micro-tumor drug testing and migration assay. The critical operation step in our methodology is direct pipette dropping the pre-cultured or pre-treated tumor spheroids on the bottom well, where subsequent image-based analysis could be performed seamlessly (see Fig. 1A, E). Notably, this dripper-like approach eliminates the need for tiresome retrieval and transferring program, which was often inevitable in downstream cell spheroids analysis. We demonstrated four typical tumoroid-based assays by implementing our strategy: (i) Anti-tumor drug screening; (ii) tumor dissemination and invasion on/inside ECM environment; (iii) tumor spheroids transendothelial migration; (iv) heterotypic spheroids fusion assay (Fig. 1A). All these assays can be performed on with a single 3D-phd device combined with a standard culture plate without other recover or grafting procedures, enabling conciseness for downstream analysis. Beyond imaging analysis, our array allows independent qPCR assay for single spheroid directly after produced (Figs. S8 and 9). Figure 1E shows a typical MCF-7 tumor spheroid generated on our 3D-phd device after two days of cultivation. We found that other than 2D culture, the F-actin filaments (phalloidin-rhodamine stained)  were bundled in cell-cell junction inside cell packed tumor spheroid. Also, the results hint that the smaller spheroid (~200 µm) allows dye penetration comparing with large aggregate (Fig. S10).

To validate the flexibility for the fast prototyping of our method, we designed a double nozzles SCS device to study the merging dynamics between different tumor spheroids. The design allows two hanging drops to coexist at SCS (Figs. 1F, and S4), and each dew can carry specific cells for culturing certain tumor aggregate. Consequently, these hanging droplets can be flushed down into the same U-shaped well (Sumitomo ultra-low attachment 96-well plate, Japan), bringing two aggregates interacted with each other. We utilized pre-stained MCF-7 (CellTracker Red) and MDA-MB231 (CellTracker Green) breast tumor cells to demonstrate this proof-of-concept. The fusion process of two different tumor spheroids can be observed after *in-situ* generation and direct dripping down (Fig. 1F). This double hanging-drops per well format realized at least three superiorities compared with the single hanging-drop. Firstly, different cell spheroids can be accurately paired in a one-to-one manner. Secondly, this format eliminates the need for manipulating spheroids or hanging-drop device to bring the heterogeneous aggregates together. Additionally, the whole process of culturing and investigation of heterotypical 3D tumorigenesis can be integrated within a single plate. To our knowledge, such a double hanging drops setting-up has not been presented in any commercialized platform.

To reiterate the distinction of our strategy, we analytically compared this method with the commercialized platform in Table 1. From the table, it can be seen that our approach shows advantages in terms of promptness, flexibility, and variety of assays without recovery. Besides the merits, it is also noteworthy that the commercial device still owns a certain degree of superiority on spheroids uniformity and reproducibility. However, this side by side examination underlines that the 3D-phd approach is more suitable for a regular laboratory that demanding fast performance of cell aggregates-based assay without uneasy manipulation process such as single spheroid retrieval.

**Table 1:** Summary of comparison between commercial product (GravityPlus™ Hanging Drop Plates) and our 3D-phd device method comparison.

	Commercial product	3D-phd
Price (User end)	USD 90/plate	USD 0.30/plate
Format	384/96 wells	384/96/arbitrarily
specification	special plate	standard
Delivery time	2 months in China (mainland)/2 days in the U.S.	1 hour
Subsequent analysis	Indirect (retrieval required)	Direct analysis (pipette down)
Advanced applications	N/A	Double hanging drops per well (1:1 pairing of different spheroids)

### Characterization of tumor spheroids generation

We first confirmed that there is no cytotoxicity of the polylactide used in our 3D printer (Fig. S11). To optimize our 3D-phd design, we investigated the spheroids formation in different design formats. Using 1500 cells per drop, we compared the 3D-phd device with and without the holding ring structures (Table S1)^26^. The results suggest that the holding ring structure can significantly enhance the hanging drop stabilization which consequently improved the cell spheroids yield (from 63 ± 11% to 97 ± 2% for 96 well formats, and 54 ± 10% to 93 ± 4% for 384 well formats). Also, we found that the holding ring can also support the spheroids' uniformity, inferring the optimization of the rapid prototype is essential for the 3D printed device.

**Figure 2:**
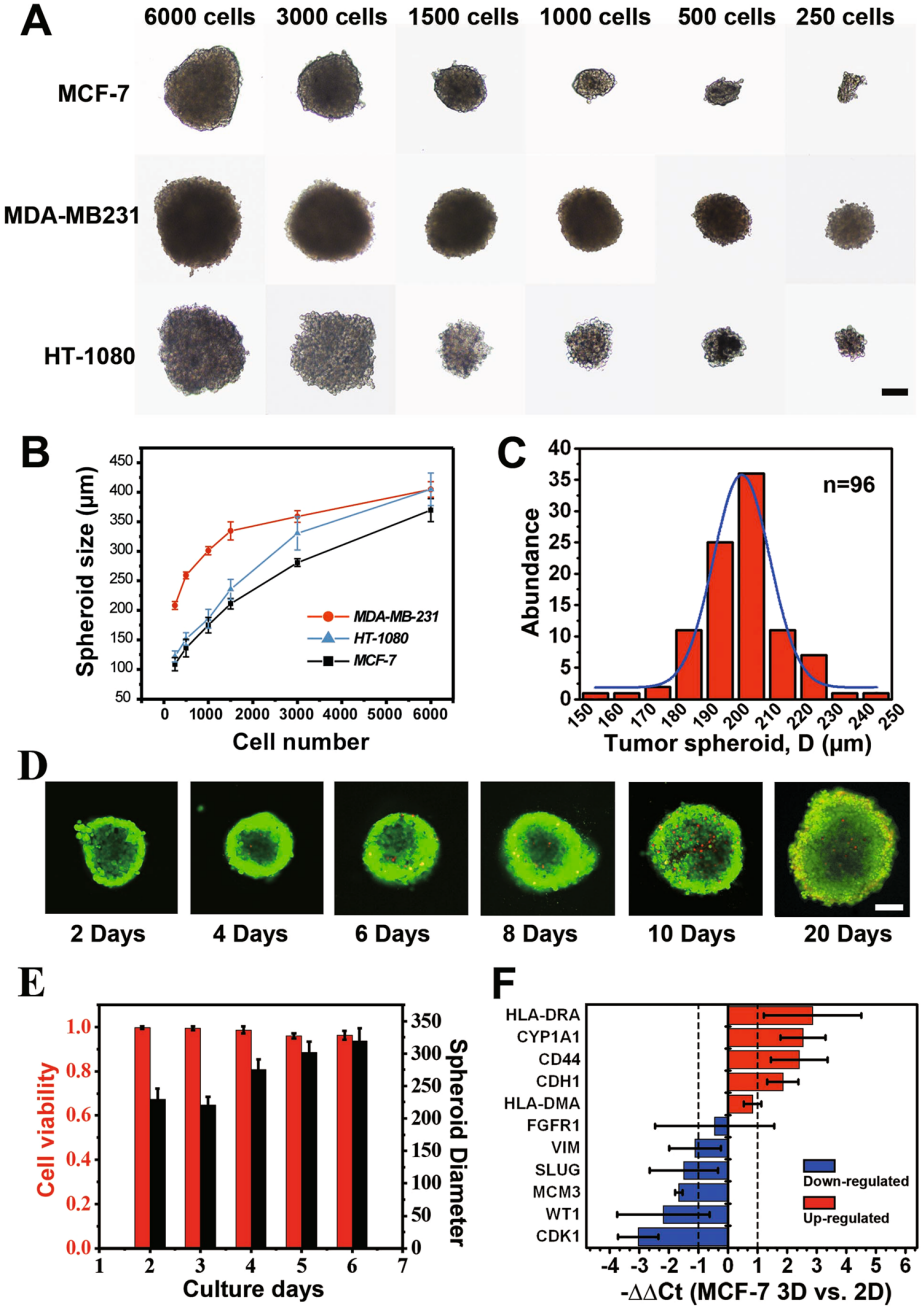
Characterization of tumor spheroids generation. (**A**) Micrographs of HT-1080, MDA-MB-231, and MCF-7 cell spheroids with different cell concentrations after cultured in 2 days. All spheroids were cultured in a 30 µL hanging drop. (**B**) The diameter of HT-1080, MDA-MB-231, and MCF-7 cell spheroids over two days culture began with a different number of seeding cells. n ≥ 9 for each cell line. (**C**) The size distribution of MCF-7 spheroids cultured on single 3D-phd for two days. The MCF-7 cells were loaded at a density of 5 × 10^4^ cells mL^−1^, and the medium was changed at 24 h after cell seeding. The average size of spheroids on this single device was 205 ± 20 µm (n = 96). (**D**) Series of confocal images of live/dead double-stained cell spheroids over 20 days. Scale bar is 100 µm. (**E**) Histogram analysis of cell viability and spheroid size changing. MCF-7 spheroids with 1500 cells/hanging drop were investigated over six days. n ≥ 5 for each bar. (**F**) Gene expression comparison between the 3D spheroid and 2D monolayer of MCF-7 cells. Relative expression (−∆∆Ct) of 11 genes has been profiled, and GAPDH was served as an internal reference gene. n = 6. Red and blue histograms indicate up- and down-regulation, respectively. Fold change (2^-∆∆Ct^) > 2 was chosen as a criterion for a significant difference.

We then investigated if well-controlled tumor spheroids can be obtained from the 3D-phd-array with long-term maintained cell viability. As proof-of-concept demonstration, to control the micro-tumor size, 30 µL of cell suspensions of different cancer cell types (breast adenocarcinoma MCF-7, MDA-MB-231, and fibrosarcoma HT1080) with different density (6000, 3000, 1500, 1000, 500 and 250 cells/drop) was seeded in the 8 × 12 printed array for characterization. The results show that the size of the micro-tumor cluster was highly correlated with the cell seeding density (Fig. 2A), indicating this device is capable of growing size-controllable cell spheroids using multiple cancer cell lines. Also, it is noteworthy that the size of cell aggregates is varied by using different cell lines. At low cell density (below 1500 cells per drop), MDA-MB231 (MM231) formulated a larger tumor size (280 µm in diameter at 1500/drop) than MCF7 (230 µm in diameter) and HT1080 cells (200 µm in diameter). As shown in Fig. 2B, the MM231, and HT1080 cells formed a looser and larger aggregate comparing with MCF-7 at the same cell amount. This result indicates that compared with the other two cell types, MCF-7 was tending to congregate more tightly after cell spheroid formed. Besides, we found that tumor-endothelial hybrid cell spheroid can also be formed on 3D-phd (Fig. S12).

We then explored the size distribution to verify if our device exhibited an excellent ability to produce size uniformed MCF-7 cell spheroids (Figs. 2C, S12). On one single device (96-well format), the average size of the MCF-7 cell spheroids was 205 ± 20 µm (n = 96) in diameter at an initial seeding density of 5 × 10^4^ cells mL^−1^. A micro tumor of 200 µm in diameter estimated ≈1000 cells/single spheroid. After seeding the tumor cells in 3D-phd, compact cell aggregates generated in 12~24 h, with a nearly 100% success rate in each hanging drop site (Table S1, Supplementary Video S1). Based on size measurement, we statistically checked the reproducibility of spheroids generation with three independent printed arrays (96-well format), as shown in Fig. S13. We next scanned the viability during growth on the 3D-phd device to substantiate that our method can enable a long-term microtissue culture in hanging drop. The live/dead staining reveals that cell viability remains relatively high for 20 days hanging mode culture on our printed device (Fig. 2D), indicating that the medium changing is necessary for culturing cell spheroids on the array. Besides, statistical results also illustrated the dynamic changes of spheroids size, indicating a fact that the micro tumor was not continuously growing after the aggregates formed. Tumor aggregate shows a self-organization process, shrinking ≈ 6–7% of its size (from 240 to 225 µm), and get into a rapid growth phase then after (from 225 to 320 µm) as shown in Fig. 2E.

Compactly growing in 3D form induces a different gene expression pattern as compared to the monolayer, because cell spheroids possess a substantial different cellular milieu that mimics more closely that of the native tumor microenvironment^27^. Therefore, we then assessed representative genes involved in tumorigenic transcription, drug metabolism, cell-cell junctions, and adhesion in the MCF-7 breast cancer cell line by using qPCR analysis (Fig. 2F). The expression of genes encoding junction and adhesion proteins was markedly upregulated compared with that in 2D monolayer culture, including E-cadherin (CDH1, 3.7-fold), CD44 (5-fold), and HLA-DRA (6.4-fold). On the other hand, the expression of mesenchymal related genes like WT1, SLUG (SNAI2), and VIM was downregulated in 3D tumor spheroids^28^. This expression pattern indicated a higher epithelial cellular statement and overall tighter cell-cell contact compared with the planar culture where cell only gets a limited bidirectional communication. Also, the metabolizing enzyme-coding gene CYP1A1 (5-fold) was enhanced while the proliferation of relevant gene CDK-1 (8-fold lower than 2D) and MCM3 (4-fold decreased than 2D) were diminished in cell cluster compared with cell monolayer (Fig. 2F). Taken together, these results disclose a hint that in MCF-7 self-organized aggregates, with promoted metabolization and cell-cell connection, cells are prone to stay at the quiescent and epithelial-like state rather than mesenchymal phase.

### Drug resistance analysis

**Figure 3:**
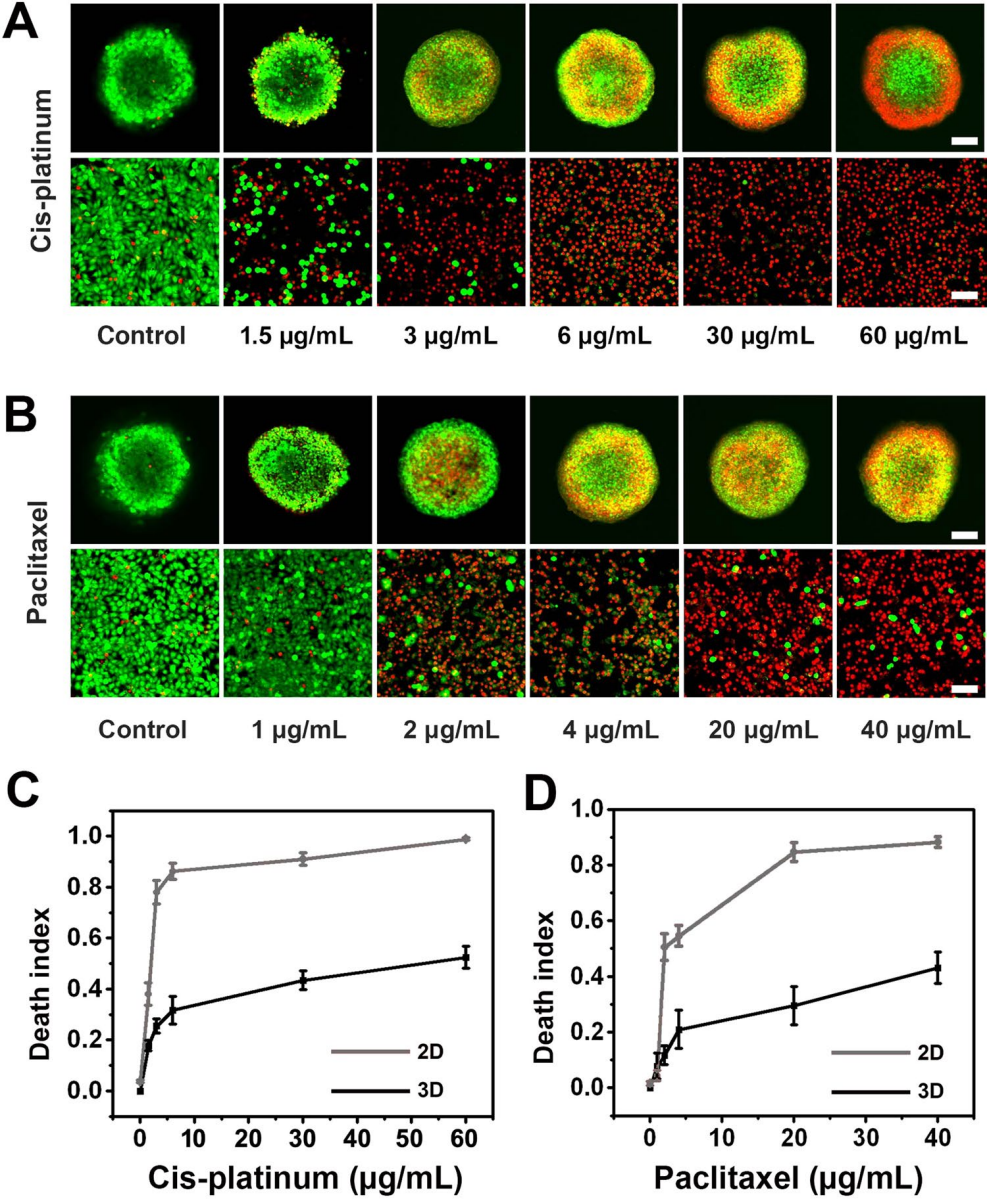
Drug resistance analysis. (**A**) Representative confocal images (live/dead double staining) for dose-dependent drug screening of MCF-7 spheroids and 2D monolayer with cis-platinum treated for 48 h on 3D-phd. (**B**) Confocal images for live/dead double staining of different concentrations of paclitaxel test in 3D-phd formed spheroids versus conventional monolayer culture. (**C**) The dose-dependent cell death index plot with different concentrations of cis-platinum. (**D**) Cell death index plotting with different concentrations of paclitaxel under 3D culture and 2D culture, respectively. For each spheroid, the death index was the average measurement from 10 confocal images along the z-axis. The death index was obtained from 10 cell spheroids at each drug concentration (**C**,**D**, n = 10). Scale bar is 200 µm.

To determine the capability of our 3D-phd platform, we compared the chemoresistance of 3D spheroids with 2D culture upon being subject to the effect of paclitaxel and cisplatin^29,30^, two commonly used chemotherapeutic drugs. We analyzed cell viability using the confocal image-based analysis tool to evaluate the dose-dependent response of the micro tumor spheroids to these anticancer drugs. Figure 3A,B shows series of live/dead staining fluorescence images after drugs were applied to 2D and 3D cultures for 48 h at different concentrations up to 40 and 60 µg/ml for paclitaxel and cisplatin, respectively. Regarding tumor aggregates, we found that cell death events mainly occurred in the peripheral region (Fig. 3A,B), indicating that the chemical compounds were challenging to get into the micro-tumor. As a result, the cell death index in two different models generally shows the dose-dependent manner when treated with cis-platinum and paclitaxel (Fig. 3C,D). Cell spheroids generally displayed a higher drug resistance to anticancer drug treatment in comparison with the 2D cell culture. In terms of cis-platinum, compared with 3D tumor spheroid (25%), 2D monolayer exhibit considerable high death index (78%) at the dosage of 3 µg/mL, as shown in Fig. 3C. The calculated IC50 value is 51.2 µg/ml for tumor cluster, whereas considerable low for planar cancer cells (1.9 µg/ml).

Similarly, for another anticancer drug paclitaxel, the clumpy tumor shows a high IC50 dose (121.46 µg/ml), which is over 30 times in magnitude compared to those dish-cultured cells (4.19 µg/ml). Collectively, based on imaging analysis (z-axis sum up), the dose-dependent curve naturally suggested that tumor spheroid is generally more resistant to an anticancer drug than 2D culture. To substantiate our viability measurement, we also compared our confocal based assay with luminescent cell viability assay (CellTiter-Glo, Promega). Under the same condition of drug treatment (series of cisplatin concentration), our multiple confocal z-axis viability measurements were consistent with the commercialized kit (Fig. S14).

The above observed chemoresistance in tumor spheroids could be attributed to several factors, including (i) the slower proliferation rate within spheroids, (ii) diffusional limitations in the three-dimensional spheroid architecture compared to monolayer, (iii) microenvironmental changes that impede drug penetration into tumor core in 3D spheroids. Therefore, our methodology can analyze anticancer drug-responsive discrepancy between 2D and 3D tumor model, underscoring the great importance of 3D culture platforms in drug screening and development, which may bridge the gap between pre-clinical experiment and conventional assay.

## Tumor Spreading and Inhibition Analysis

**Figure 4:**
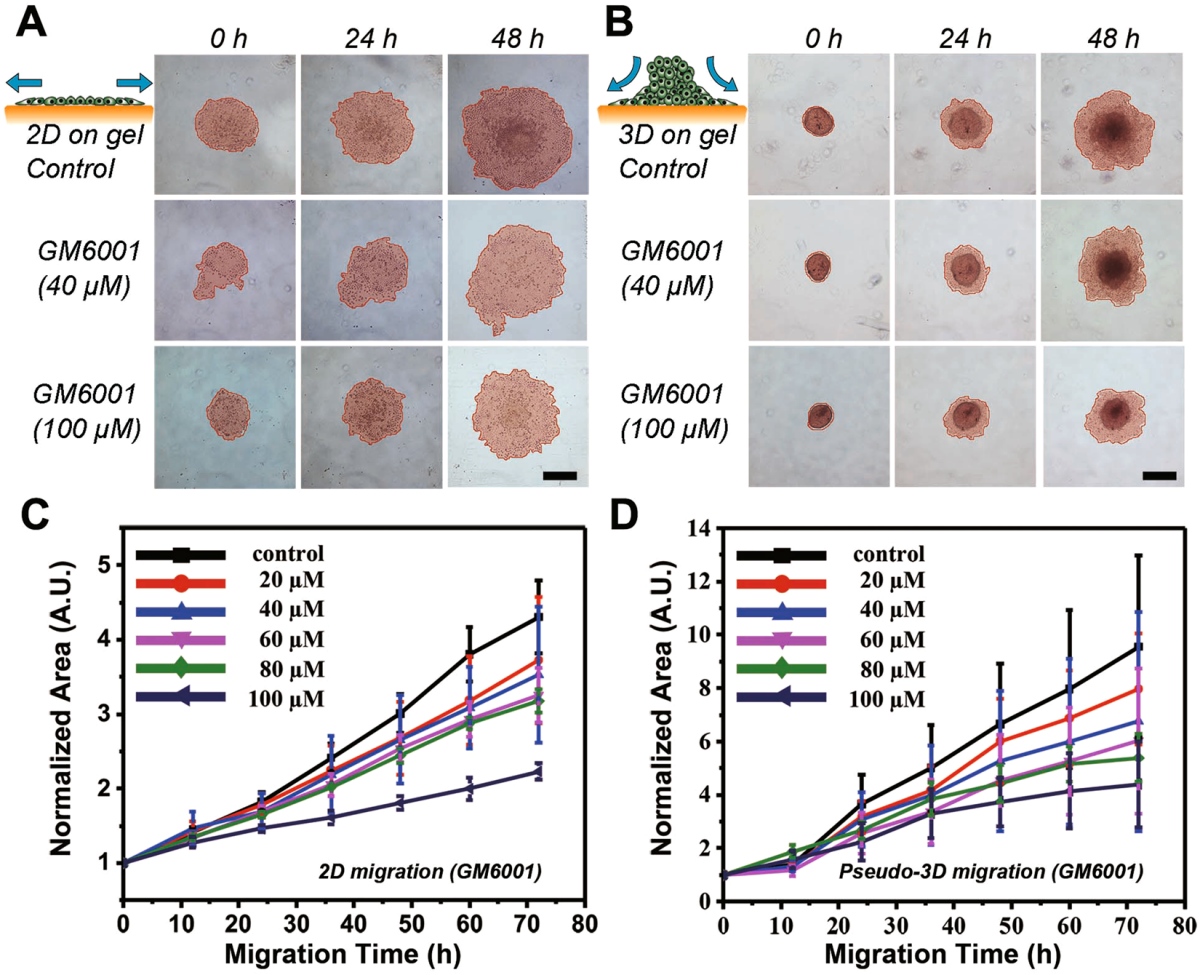
Schematic diagram and tumor cell migration characterization over 48 h in conventional monolayer and pseudo-3D conditions. (**A**) Schematic illustration of 2D cell migration and time-lapse micrographs for monolayer tumor cell migration under the treatment of GM6001 at different concentrations. The cell occupied area is indicated with salmon color. (**B**) Schematic illustration of 3D cell aggregates dispersion on the collagen gel surface and time-lapse images for cell spheroids migration under treatment of GM6001 at different concentrations. The cell occupied area is indicated with salmon color. (**C**) Quantitative plot for 2D cell migration at different concentration inhibitor conditions (N = 12). (**D**) Migration curve for pseudo-3D cell aggregates at different concentrations of GM6001 treatment (N = 12). Scale bar is 200 µm.

We next compared the 3D cell aggregates dissemination on an ECM coated substrate with 2D cell monolayer migration under specific inhibition factor. GM6001 (Ilomastat, or galardin), a matrix metalloproteinase inhibitor, has been chosen as an anti-motile test agent, was applied in both cases to mimick tumor cell invasion process with inhibition on cell motility^31^. In Fig. 4A, micrographs at different time points show the dose-response profile that the collective migration has been proportionally inhibited as the increase of inhibitor concentration on both 2D monolayer and 3D dispersion. In consequence, for precisely depicting cell migration dynamics, we measured the normalized area of cell-occupied region plotted for time-series by analyzing time-lapse images when the cancer cells were migrated through ECM coated surface for three days. As expected, the cell migrated over the time results exhibited slower migration with a higher GM6001 dose in both 2D and 3D conditions, as shown in Fig. 4B,C.

Besides, the quantitative inhibition curve suggests that the aggregate moves faster than 2D cultured tumor cells indicating there is a radically different mechanism for cell migration between 2D and 3D invasion assay. With no drug addition, the tumor spheroid dispersion region was doubled in 15 h, and increased fourfold in 25 h from the initiation while the 2D gained the same area ratio in 24 h and 64 h, respectively (Fig. 4B,C). This phenomenon is, at least, partly caused by the relocation of the cells from the cell cluster top to the peripheral edge bottom and thus makes cell number significantly increased during collective dissemination on ECM coating planar substrate. The collective invasion has been significantly blocked in tumor monolayer (migration index was a 2-fold increase from the beginning). In contrast, GM6001 was not that much considerably affect tumor spheroid (migration index was nearly 4-fold climbed) at the highest dose (100 µM). A possible rationale of this noncompliance is that as a metalloproteinase depressor, GM6001 was primarily targeted on cell-matrix interaction rather than cell-cell dissemination. The deputed cell transmission from inside and top, contributing the invasion index for 3D tumor. Thus, our results implied that even in a model as simple as cell migration, the influential factors in the 3D tumor niche could be more complicated than traditional inadequate 2D *in vitro* assay due to the other competitions between cell-cell and cell-substrate adhesion.

Moreover, it is notable that the whole experiment can be accomplished in our 3D-phd array, including tumor spheroid generation, micro-tumor harvesting, anchoring on the ECM coating bottom, and *in situ* analyzing of cell dissemination. This operation integrity is essential because it provides not only a shortened procedure for efficient analysis on one single device but also the elimination of any possible contaminations or sample loss during the spheroids' transferring process. As shown in the supplementary information (Video S2), after tumor spheroids have been cultured on a printed array for 48 h, the hanging drops were pipetted down (50–100 µL culture medium depending on the assay) into 96 well directly.

### 3D tumor metastasis study

**Figure 5:**
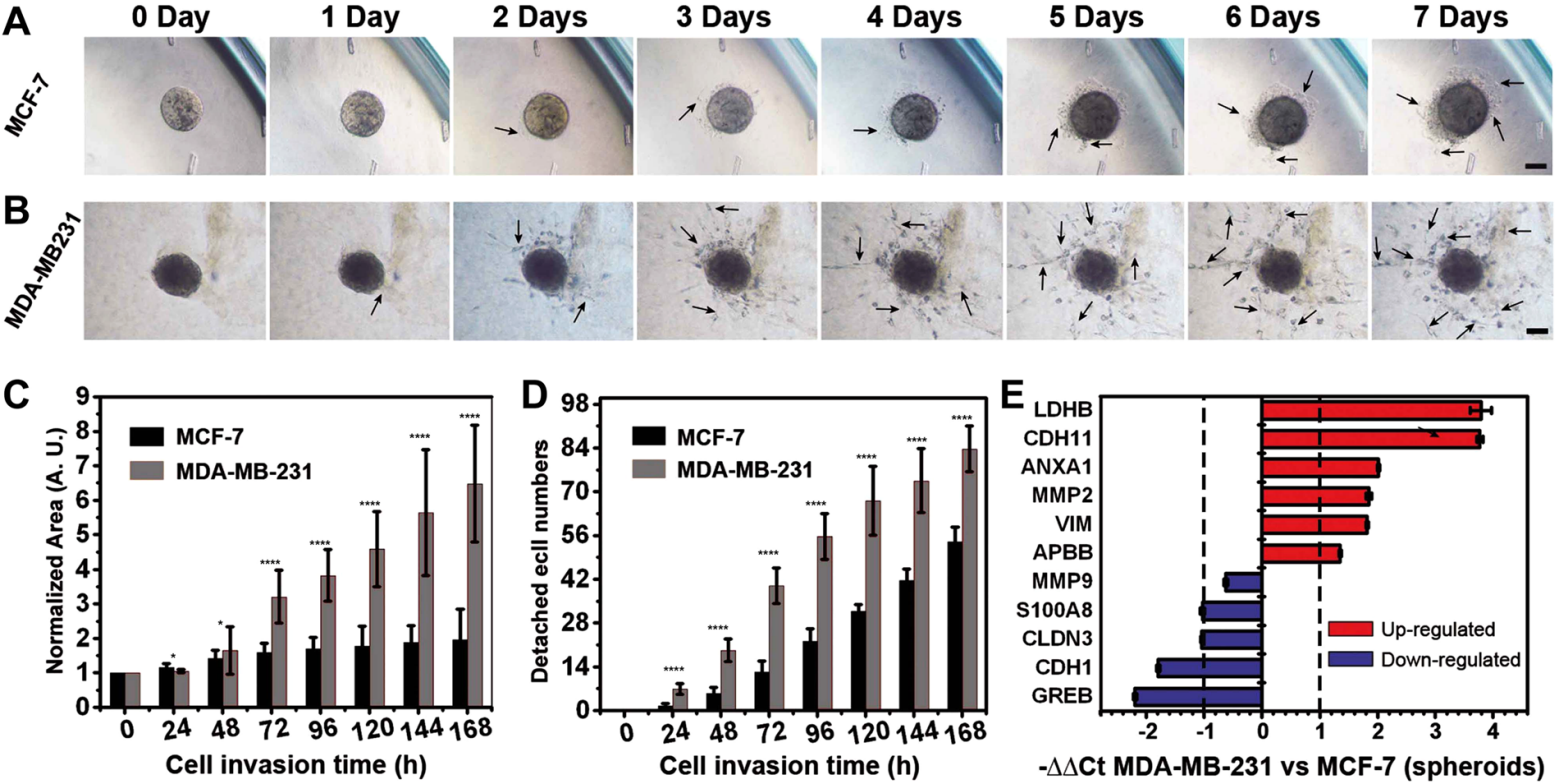
3D cell spheroid invasion assay. (**A,B**) Time-lapse images for different cell spheroid invasion inside collagen gel. These images show significant phenotypical differences between less-malignant cell type (MCF-7) (A), and invasive cell type (MDA-MB-231) (**B**) throughout seven days. The analysis of the invasion area (**C**) and detached leading cell numbers (**D**) illustrate the different metastatic behaviors in these breast cancer cell lines. Statistical significance was calculated via the T-test. *P < 0.05, **P < 0.01, ***P < 0.001, ****P < 0.0001. (**E**) The gene expression analysis in those two types of cell spheroids, respectively. The housekeeping gene GAPDH was used for normalization (N = 6). Red and blue histograms suggest up- and down-regulation, respectively. Fold change (2^-∆∆Ct^) > 2 was chosen as a criterion for a significant difference. Scale bar is 100 µm.

Consequently, we next adapted our 3D-phd platform for investigating the kinetic invasion of clumpy cell aggregates within an extracellular matrix enriched environment. Our 3D-phd-array can provide a seamless resolution because the spheroids generation and cell migration study can be performed on a single device where standard liquid pipetting is exclusively the only procedure that needed during the whole experiment. To validate if our method is capable of achieving this, after two days of spheroids culture on a 3d-phd device, we analyzed the metastasis of different breast tumor cell spheroids directly by dropping cell spheroids into the bottom well with ECM gel and measuring cell migration process dynamically. Time-lapse images suggested that we can achieve long-term study (14 d) of the tumor invasion inside the ECM environment (Fig. S15). We also examined if cell proliferation occurs even the whole spheroid embodied in the collagen gel. In Fig. S16, the result shows that most cell mitosis (Ki-67 positive) events can be found at the exterior parts of for 14 days spheroid metastasis, indicating the existence of a quiescent core in each tumor spheroid. Using our 3D-phd, we next compared the invasiveness between two types of breast tumor cells, MM231 and MCF-7. Figure 5A,B illustrated that MM231 spheroids were gradually invaded into the gel and migrated either in forms of cellular sprouts or individual cell which detach from the origin spheroids, whereas MCF7 spheroids show dragging collective migration rather than metastasis. In the first two days, little protrusions were observed, and subsequently, elongated protrusive invadopodia-like cells were significantly presented around MM231 clump (2–7 days, Fig. 5B). Even at five days, these pseudopodia multi-cell structures can scarcely be detected in the peripheral-zone of MCF-7 spheroids (Fig. 5A). To characterize the cell metastasis inside collagen gel, we employed cell projected area and isolating cell number as two criterions for measuring invasion quantitatively. After 48 hours, the MM231 invasion area was dramatically increased (normalized migration region expanded two times after 72 h, and three times after 168 h), whereas the MCF invasion area consistently reached a plateau, as shown in Fig. 5C. We also analyzed the number of single-detached cells that play a leader role in spheroid-based metastasis. The results strongly suggested that compared with the MCF-7 3D tumor, the MM231 aggregates are more invasive (Fig. 5D).

Importantly, fewer cells were able to detach from the MCF-7 spheroid and migrate, implying the morphogenic impact of transcriptional regulation between these two types of tumor cell lines. Beyond the morphological observation, the profiling of gene expression is particularly required for studying specific pathways. The phenotypical-to-molecular comprehensive characterization allows a better understanding of tumorigenesis. Therefore, we next determined the expression level of corresponding functional genes (LDHB, CDH11, ANXA1, MMP2, VIM, APBB, MMP9, S100A8, CLDN3, CDH1, and GREB) in tumor aggregates of both cell type for studying the regulatory differences during metastasis (Fig. 5E). Among these genes, GREB1 (an estrogen-responsive element) was used to distinguish MCF-7 and MM-231 cells (a triple-negative breast cancer cell line). We confirmed six candidate genes (LDHB, CDH11, ANXA1, MMP2, VIM, and APBB) that were significantly upregulated whereas four genes (S100A8, CLDN3, CDH1, and GREB1) out of 11 total genes (>2-fold changes) were down-regulated in malignant MM-231 spheroid compared with less malignant MCF-7 tumor cluster. Notably, in MDA-MB 231 clump, the gene expression pattern shows mRNA level of epithelial markers Claudin-3 (CLDN3) and E-cadherin (CDH1) were evidently decreased (2-fold and 3-fold respectively), while the mesenchymal related factors E-cadherin 11 (CDH11) and Vimentin (VIM), were upregulated (16 fold and 4.5 fold, respectively) compared with non-invasive MCF-7 spheroids^32^. This transcriptional changing agreed with recent studies that described that, besides the typical epithelial to mesenchymal transition (EMT) marker vimentin, EMT is often accompanied by the expression of CDH 11 and diminished expression of E-cadherin and Claudin-3^33,34^. Moreover, the up-regulation of Annexin A1 and LDHB in our results are consistent with literature that depicted enhanced TGFβ/Smad signaling (induced by Annexin A1) and aerobic glycolysis pathway in EMT-switched basal-like breast cancer cells^35,36^. Importantly, we found other cancer progression factors are also tightly associated with different phenotyping during 3D tumor metastasis. For example, the mRNA expression of matrix metalloproteinases-2, one of the principal mediators of the alterations observed in the microenvironment during cancer progression^37^, is highly enriched (4-fold increase) in MM-231 cell spheroid. In contrast, the MMP-9 level was not distinguishable between these two types of spheroids, indicating a delicateness and possible controversial pattern of protein family members.

### 3D tumor transendothelial migration study

**Figure 6:**
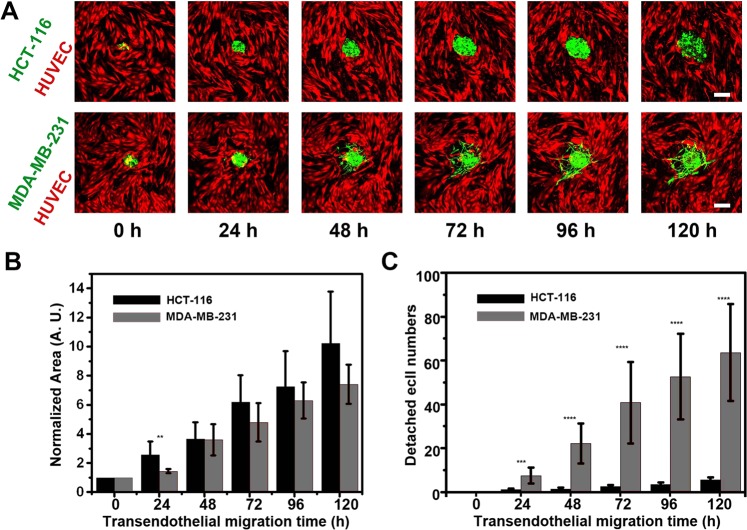
3D tumor spheroid transendothelial migration assay. (**A**) Time-lapse confocal images for different cell spheroids transendothelial migration inside collagen gel (120 h). These tumor spheroids-based transmigration micrographic images indicated the phenotypical differences between human colon cancer cell line HCT-116 (HCT-116-eGPF, green) and human breast cancer cell line MDA-MB-231(MM231-eGFP, green). Human umbilical vein endothelial cells (HUVECs-tdTomato, red) were used for simulating the endothelium layer on the surface of previously coated collagen in the bottom well. (**B**) The plot of different types of cell aggregate projection area on the interface of tumor-endothelium versus time (n = 6). (**C**) The quantitative analysis (n = 6) of detached aggressive cell numbers during tumor transendothelial migration (TEM). Statistical significance was calculated via the T-test. *P < 0.05, **P < 0.01, ***P < 0.001, ****P < 0.0001. Scale bar is 100 µm.

We next sought to extend our methodology to dissect tumor spheroid transendothelial migration (TEM) with our 3D-phd device. To simplify the fabrication of the endothelial layer, we cultured the HUVECs on the collagen-coated culture well for 24 hours. After tumor spheroids have been produced, the tumor containing 3D-phd array was translocated onto the HUVECs plate. Due to the merit of dripping down tumor spheroids into the bottom well with gel solution, the tumor spheroids TEM could be continuously quantified in a 3D milieu using confocal time-lapse. Like in gel metastasis assay, we can see that the MM231 spheroid TEM represents a more malignant phenotype compared with the colon cancer cell aggregate. The peripheral MM231 cells turn out to dissociate themselves from tumor clump and represent a stellate morphology, whereas the colon cancer cell HCT-116 cells are prone to be disseminated collectively (Fig. 6A). Interestingly, we also figured out that at the interfacial tumor-endothelium plane, these two cancer cell lines present a comparable collective TEM phenotype (Fig. 6B, no significance). On the other hand, the statistic measurement of dispersed leader cells (more than 60 cells detached vs. less than ten cells after five days, respectively) still underlines the invasiveness of MM231 tumor cells (Fig. 6C).

## Discussion

Conventional ways to generate 3D tumor spheroids focused on creating an effective method to produce a significant amount of size-controllable cell aggregates. However, if we rethink, readdress, and reassess the whole scenario in both preclinical cellular assays and necessary biological investigation, a conclusion would be quite inferable: the capability of easy recovery and down-stream analysis of the cultivated cell lump is conclusively essential for *in vitro* 3D cell culture technique. Moreover, it is highly desirable to recapitulate 3D tumor *in vitro* and harness them to execute the elaborate assay in a fast and cost-effective way. Thus, we determined to address this technology gap by developing a simple approach that can leverage a desktop 3D printer to fulfill lab-routine generation of cell spheroids, which can be used for downstream assay directly.

We describe a 3D printed hanging-drop dripper (3D-phd) for cellular spheroids generation that allows a long-term culture of self-organized-spherical cell clusters, drug testing analysis, profiling of gene expression, and morphological phenotyping of invading tumor cell aggregates inside extracellular matrix gel. For biological researchers, it is vital to have an inexpensive and rapid prototyping method for performing a biomedical assay for the first time they get testing samples. Due to the accessibility of “STL/OBJ” file on any source sharing website, our platform could be easily replicated by bioengineering laboratories equipped a desktop 3D printer, avoiding microfabrication and any further manufacturing^38^. By implementing this strategy, different submillimeter tumors have been obtained and analyzed on the device, which could be printed out within one hour. We show that by employing 3D-phd array, we can produce tumor aggregates with high uniformity, viability, flexibility, and controllability.

Moreover, we demonstrated that the heterogeneous spheroids pairing, and fusion can be accomplished on a printed double-nozzles 3D-phd array. Such a format bypasses the need for transferring different spheroids on a different device, showing the advances of leveraging additive manufacturing for fabricating biological assay tool. This double hanging drop system has not yet available in any commercialized platform. In our approach, the direct dripping-down allows us to perform multiple subsequent assays without specific recovery procedures, significantly smoothing aggregate-based cancer study. Expression profile infers that the metabolism, cell-cell junction, and epithelium associated genes were elevated in the 3D tumor. Anticancer drug testing has been shown between 3D-phd and planar cell culture group to validate the capability of our method. The result shows considerable differences in resistance to extrinsic cytotoxicity in hanging drop tumor spheroids compared to a 2D monolayer.

Tumor cell dissemination plays a pivotal role in cancer development because the migration of cancer cells from a primary tumor is an essential step for cancer progression. More importantly, the cell may leverage different mechanisms in different micro-environments. For a tumor to metastasize, cells will firstly invade in ECM which mediates cell polarity, intracellular signaling and supporting in migration^39^. To facilitate tumor migration, the local ECM must be remolded, creating an environment conducive to tumor survival and development. Therefore, quantitative analysis of cancer cell migration from a solid tumor could potentially provide a way to understand such invasion and metastasis events. Conventionally, *in vitro* assays for tumor migration are often reckon on measuring cell healing^40^, cell patterning^41^, and Boyden chamber-based cell migration assay^42^. All these approaches are incapable of mimicking tumor development because of their intrinsic limitation, the cell monolayer, which may lead to a short recapitulation of *in vivo* metastasis conditions. In terms of 3D tumor-based assay, previous existing methods with the incorporation of spheroids making and 3D migration assay are also limited due to several hurdles, including difficulties for retrieving aggregates from microwells, transferring aggregates to ECM niche, and absence of analyzing gene expression relevant to tumor invasion. We thus applied our 3d-phd approach to dissect the tumoroid-based dissemination and metastasis onto/inside the ECM (collagen gel) without complex retrieval operation. Results exhibited that the metalloprotease inhibitor GM6001 can impair cell migration on the ECM surface with a dose-dependent manner. The metastatic assay shows the malignant phenotype of MM-231 cells compared with MCF-7 cells when aggregate has been embedded in ECM gel. By comprehensive analyzing the gene expression between two types of breast cancer cell, we disclosed the correlated EMT-correspondent regulation in MDA-MB231 tumor aggregates, further implying that this technology is not only can quantitatively decipher morphological conversion, but also unveil molecular oncologic pathology changeover.

The metastatic cascade requires dynamic alterations of the interactions among the tumor cells and of tumor cells with other cells or the extracellular matrix. During metastasis, the entry of tumor cells into the vascular system (intravasation) and escape from circulation (extravasation) to distal tissue are critical steps which involve tumor-endothelial interactions^43^. These processes require the disruption of the endothelial barrier where the tumor cells invade into endothelium and transmigrate across the endothelial border. Understanding the detailed mechanism behind tumor intravasation and extravasation remains challenging. To address this, researchers must recapitulate the tumor-endothelial model in the 3D scenario *in vitro*. Several microfluidic-based approaches have been developed to analyze tumor-endothelial cell interactions^44–46^. However, these methods are based on sophisticated microfabrication and require careful manipulation to construct the 3D interfaces between tumor and endothelial cells. Therefore, besides 3D invasion assay, we extended our methodology to dissect spheroid based transendothelial migration where collagen gel provides an *in vivo* like milieu for investigating metastatic tumor dynamics. Due to the merit of prompt dripping down tumor spheroids into the bottom well with HUVECs, tumor invasion throughout the endothelial layer could be continuously quantified based on our 3D-phd approach. To our knowledge, this is the most straightforward demonstration that realized tumor spheroids based transendothelial migration assay with quantitative measurement.

Taken together, we have demonstrated that our device can seamlessly accomplish series of biomedical assays including tumor cluster generation, one-to-one pairing and merging between different spheroids, imaging analysis on different anticancer chemicals, cell desperation either onto or inside ECM environment, and gene expression mapping to disclose possible mechanism behind different tumor invasion phenotype. Compared with other methods, direct dropping down the micro-tumor onto 96 well bottoms provide an ultra-easy way to perform 3D tumor migration analysis because it allows us to analyze the metastatic, dissemination, and transendothelial dynamics with a standard *in situ* culture and imaging. The proposed methodology could also be easily extended to apply multiple organ-on-chip engineering, assess the drug screening on co-cultures, and study the effect of the external environment on tissue behavior. Therefore, we envisioned that this 3D-phd methodology might open a possibility for switching the current “lab-on-chip” scenario to future “lab-on-cloud” era when one can routinely conduct biological assays by downloading the device prototype from an open-source community.

## Materials and Methods

### 3D printed hanging-drop dripper (3d-phd) design and manufacture

We designed the prototype of our 3D-phd device by using 3DS Max 2015 (Autodesk). Three independent formats have been developed to fit a standard 96-well, 24-well, or 384-well plate. A bench-top 3D printer (MakerBot Replicator II, NY, USA.) was used to fabricate our device using PLA filament. For a typical 96-well format, each cell spheroid culture site (SCS) consists of three parts. (i) the 3/4 circle skirt tray (5.7 mm diameter) with a through tunnel (1.6 mm in diameter) at the center of the plate; (ii) the cylinder ring structure under the beneath of the skirt plate base; with 1.6 mm inner tunnel, cylinder wall (0.95 mm thickness) and 1.5 mm in height; (iii) In the bottom of the cylinder, there is a holding ring structure (same outer ring edged with the upper cylinder, 3.5 mm) with 0.5 mm cylinder wall thickness to hold the cell suspension and stabilized medium. The detailed information of design can be found in the supplementary material (Figs. S1–4).

### Cell and spheroid culture

MCF-7 and MDA-MB-231 human breast cancer cells, HT-1080 human fibrosarcoma cells are provided from Stem Cell Bank, Chinese Academy of Sciences. HCT116-eGFP (human colon carcinoma) stable cell line was a generous gift from Prof. Jianbin Wang (School of Life Science, Tsinghua University). MDA-MB231-eGFP cell line was purchased from Beijing Dingguo Changsheng Biotechnology. These cell lines are cultured in Dulbecco’s modified Eagle’s medium (DMEM, Gibco) supplemented with 10% v/v Fetal Bovine Serum (Gibco), 1% v/v Pen/Strep, 1% v/v glutamax, and 1% v/v non-essential Amino Acids (Gibco). We cultured HUVEC-tdTomato cells in Endothelial Cell Medium (ECM, ScienCell) supplemented with 5% v/v Fetal Bovine Serum (FBS, ScienCell), 1% v/v endothelial cell growth supplement (ECGS, ScienCell), and 1% v/v penicillin/streptomycin solution. Cell suspensions for the hanging drop experiments were in trypsin solution and subsequently neutralized with 10% FBS in DMEM. Cell density was estimated using a hemocytometer. The array was firstly incubated with 5% Pluronic F-108 (BASF) for 2 hours and sterilized with UV for 30 min before use. To produce hanging drops, 15–30 µL cell suspension solution (containing 1.2% methyl cellulose) is pipetted from the top side of the skirt plate through the access hole with the end of each pipette tip get into the access hole to guide the solution to the holding ring. To prevent water evaporation, we wrapped 96-well peripheral with Parafilm (VWR, USA). The growth media was exchanged every other day by taking 7 µL medium out from a drop, and subsequently adding 10 µL of fresh culture media into a drop. We utilized two types of stably transfected cells expressing fluorescent proteins, HCT-116-eGFP, and HUVECs-tdTomato, to generate hybrid spheroid. Simply by hanging the mixed cell suspension (tdTomato-HUVECs: eGPF-HCT116 = 3:1) on the 3D-phd array, the heterogeneous spheroids were successfully produced as shown in Fig. S12. For culturing different spheroids on double nozzle format 3D-phd array, 15 μL of cell suspension with cell number around 1000 cells per drop was carefully introduced into each nozzle and hanging drops were formed beneath the SCS. MCF-7 and MDA-MB231 cells were previously stained by CellTracker Red, and CellTracker Green, respectively. After 24 h cultured on an array, 30 μL of the medium was introduced for dripping down the drops from SCS, and a 96-well ultra-low attachment plate (Sumitomo, Japan) was used for collecting the cell spheroids at the bottom. At the time point of day1, day2, and day3, the merged heterotypic spheroids were imaged under a confocal microscope for recording the dynamic process of fusion.

### Ki 67 immunofluorescence staining

After spheroids migrated 15 days inside collagen, cells are subsequently immobilized by 4% formaldehyde for 30 min and permeated cell membranes with 0.5% Triton X-100 for 1 h. Spheroids are incubated in 1% BSA, 10% normal goat serum, and 0.3 M glycine in PBS-Tween 20 (0.1%, Tween 20, Beyotime, China) for 2 h. Next, cells are incubated with 1 μg/mL anti-Ki67 antibody (Abcam) overnight at 4 °C. After PBS wishing, we add the secondary antibody Fluor 488-labeled Goat Anti-Rabbit IgG (H + L) (Beyotime, China) at 1: 500 dilution for 1 h. Nuclei were subsequently stained with Hoechst 33342 (Dojindo, Japan), 1:250. Fluorescent images were then captured using a confocal microscope. (FV1200, Olympus, Japan). The analysis was performed by using Fiji imaging software (NIH Image, https://fiji.sc/).

### F-Actin labeling in tumor spheroid

Spheroids were washed twice with PBS, fixed with 4% formaldehyde, and permeated cell membranes with PBS containing 0.5% Triton X-100 at 4 °C for 60 min. Next, spheroids were stained with rhodamine- phalloidin (final concentration is 0.165 μM) for 30–60 min. Nuclei were subsequently stained with Hoechst 33342, at 1:500 dilution for another 1 h.

### Anti-tumor drug assay

Three different drugs (two chemotherapeutic drugs and one inhibitive cancer metastasis drug) has been chosen to test drug effectiveness in both MCF-7 cell spheroids and cell monolayer. Each drug has been dissolved in Dimethyl sulfoxide or N, N-Dimethylformamide, then aliquoted and stored at −20 °C. We optimized the concentration of anticancer drug paclitaxel (Sigma-Aldrich), Diammineplatinum (II) Dichloride (cis-platinum, Sigma-Aldrich), as the final concentration gradient is 1, 2, 4, 20, 40 μM and 1.5, 3, 6, 30, 60 μM, respectively. As described above, single spheroid was obtained by 1500 cells per drop. A drug-containing culture medium was added into a hanging drop to achieve appropriate concentration per drop by above medium exchange procedure. After 48 h drug treatment, cellular viability was characterized by using double staining with Calcein-AM (Dojindo, Japan) and Ethidium homodimer-1 (EthD-1, Abcam). The staining solution with those two dyes (with calculated final concentration) in DMEM (without FBS) was used to drop cell spheroids down to culture wells by pipetting. After 45 min at 37 °C incubation in darkness, the whole 96-wells was then centrifuged (Eppendorf 5810R, Germany) to facilitate imaging. Z-axis scanning was used to gain 10 slices for every single spheroid. For each slice, the green and red cells were separately calculated using mono-color mode. Death index was used for depicting death percentage, and average data were calculated to profile total death rate in spheroid. The death index for a single image can be calculated as below:

$$Death\,Index\,for\,each\,scanned\,layer=\frac{Red\,pixel\,area}{(Green+Red)\,pixel\,area}\times 100 \% $$

Cell monolayer for assessing anticancer drug sensitivity was performed in standard tissue culture treated 96-well plates (Nunc™ Edge, Thermofisher Scientific).

### Cell migration study on the collagen surface

We performed cell migration assay by using metastasis inhibitor GM6001. Before the migration test, aggregates were pre-cultured as described previously and checked out under a microscope. Cell spheroid was then pipetted down onto bottom well coated with collagen I. Migration was initiated by cell-attached, termed migrating precursor. Inhibitor containing medium was then exchanged with the final concentration gradient at 20, 40, 60, 80, and 100 µM, respectively. For the 2D model, cells were seeded in 96 well plates attached with a punched parafilm, which has been fabricated as patterning mold. The parafilm has been crafted by a knife (OLFA, Japan) and puncher (ø = 0.3 mm, Syneo LLC, U.S.A.) to fit with each well on 96 well plate. The parafilm was removed to form a circled cell pattern with a 0.3 mm diameter after cell has attached to the collagen-coated substrate. Migrating cells were imaged under a confocal microscope and quantitatively analyzed by using Fiji as described previously.

### RT-QPCR analysis

To determine relative gene expression difference in cell spheroids compared with conventional cell monolayer, we quantified the expression level of 11 selected genes in 2D and 3D cell culture mode, respectively. Briefly, resuspended cells or spheroids were collected by centrifugation at 1000 rpm in 2 min. RNeasy and Reverse Transcription Kit (Qiagen) was used for preparing the cDNA library from lysed cells or spheroids. To profile the relative expression of targeted genes, quantitative PCR was carried out on a qPCR system (q225, Kubo Technology, Beijing, China). GAPDH was used for normalization.

### 3D tumor metastasis in collagen gel

To recapitulate an *in vivo* microenvironment and study 3D tumor metastasis, we embedded cell spheroids in collagen gel by directly pipetting down tumor spheroid previously generated on our 3D-phd array device. The collagen gel solution was prepared from collagen type I (3 mg/mL, ThermoFisher Scientific) with 10% 10 × PBS and 1:1 mixed with 1.2% methylcellulose (Sigma-Aldrich) in the cell culture medium. After that, the pH was adjusted to 7.4 by using 1 M NaOH (Sigma-Aldrich). All solutions should be kept on ice before use. To perform gel embedding, 60 µL collagen gel solution was directly pipetted into SCS, and the spheroid containing hanging drop will fall into the bottom well. The 96-well plate was then placed into an incubator for 2 hours for gelling. Another 40 µL of the medium was then added into each well to smooth cell metastasis in collagen gel.

### 3D tumor cell transendothelial migration

To study tumor-endothelial interactions, we developed a transendothelial migration assay in our device. Briefly, 10 µL collagen gel (100 µg/mL) was added in each well and incubated at 37 °C for 2 hours for gelling. The tdTomato HUVECs were then cultured for 24 hours. A 3d-phd device which contains well-generated tumor spheroids (MDA-MB231-eGFP, or HTC116-eGFP) were transferred onto this tdTomato HUVECs plate. All tumor clumps were pipetted down onto the bottom endothelium layer with a collagen gel solution. The whole plate was then centrifuged at 2000 rpm for 5 min at 4 °C to facilitate the contact between the spheroids and the endothelium layer. The entire plate was then put into the incubator for 2 hours for collagen gelling. After carefully checked, 40 µL of culture medium was then introduced into each well. The dynamic transendothelial migration images were snapped using the confocal microscope.

## Supplementary information

Video-S1

Video-S2

Supplementary information

## Data Availability

Supplementary Information accompanies this paper.
