# Supplementary material for: A 3D Printed Hanging Drop Dripper for Tumor Spheroids Analysis Without Recovery
Source: Sci Rep. 2019 Dec 23;9:19717. doi: 10.1038/s41598-019-56241-0 (PMC6928160; doi:10.1038/s41598-019-56241-0)
Supplement: Supplementary file 3 — Supplementary information [file 41598_2019_56241_MOESM3_ESM.pdf]

Supporting Information

A 3D Printed Hanging Drop Dripper for Tumor Spheroids Analysis Without Recovery

Liang Zhao†*, Jidong Xiu†, Yang Liu, Tianye Zhang, Wenjie Pan, Xiaonan Zheng, Xueji Zhang*

Institute of Precision Medicine and Health, Research Center for Bioengineering and Sensing Technology, Beijing Key Laboratory for Bioengineering and Sensing Technology, School of Chemistry and Biological Engineering, University of Science and Technology Beijing, Beijing, China, 100083

Author Information:

*: To whom correspondence should be addressed.

zhaoliang2013@ustb.edu.cn

zhangxueji@ustb.edu.cn

†: These authors contributed equally to this work and considered co-first author.

Cell cytotoxicity assay: MTT method is a colorimetric assay to verify cell viability which also can indicate material cytotoxicity. Cells were seeded at a density of 10^5 cells/well for 24 hours to form a semi-confluent monolayer. In each cell culture well, different weight of PLA pieces (0.1, 0.2, 0.3, 0.4, 0.5, 0.6, 0.7, 0.8 g) was added into cell medium for 48 hours. After that, 10 µL MTT solution (5 mg/mL, Sigma) was added in 100 µL cell culture medium and cell were incubated for another 4 hours at 37 °C. The MTT solution was then removed and replaced by 150 µL DMSO. In order to fully dissolve MTT tetrazolium dye in DMSO, the 96-well cell culture plate was put in shaking incubator for 20 min at 150 rpm. After that, solution absorbance was measured at 490 nm absorbance with a microplate reader (Anthos 2010, Biochrom, UK). The color intensity of each well was quantified and MTT assays were repeated three times.

Commercialized spheroids viability assay: In order to verify viability measurement, we compared our results with a commercial ATP cell viability assay (CellTiter-Glo 3D reagent, Promega). Individual cell spheroid was harvested after drug treatment and washed twice with PBS to remove any extracellular ATP. Each spheroid was consequently added in 75 µL DMEM (non-Phenol red, Gibco, Invitrogen, ThermoFisher Scientific, 21063029) and transferred into opaque 96-well plates (Thermofisher Scientific, white, 236108). After 75 µL of Cell Titer-Glo 3D (Part#G9683, Promega) was added into the well, the solution was gently shaken for 5 min at room temperature to induce cell lysis. To stabilize the bioluminescent signal, plate was incubated at room temperature in dark for an additional 25 minutes and then sample signal was quantified on plate reader (Perkin-Elmer Life Sciences).

Heterogeneous cell spheroids generation: To demonstrate our 3d-phd device is also capable of generating heterogeneous cellular aggregates, we seeded 30 µL mixed cell suspension (HUVEC-tdTomato and HCT116-eGFP cells, cell percentage ration is 1:1, with 1.2% methyl cellulose in

medium) with density at 5 $\times 10^4$ / mL. After 2 days culture, we drip down the spheroids by 50 μL PBS and the plate was then centrifuged at 1500 rpm for 5 min to facilitate confocal observation.

RT-QPCR: To profile differential mRNA expression level in 2D monolayer and 3D spheroids, 11 candidate genes (WT1, SLUG, VIM, FGFR1, MCM3, CDH1, CD44, CYP1A1, CDK1, HLA-DRA, HLA-DMA) were analyzed by using real-time quantitative PCR (q225, Kubo Technology, Beijing, China). Another 11 targeted genes (LDHB, CDH11, ANXA1, MMP2, VIM, APBB, MMP9, S100A8, CLDN3, CDH1, and GREB) have been involved to measure expression pattern in MCF-7 and MDA-MB-231 cell spheroids. The operation of the qPCR assay procedure was operated as flows: 50°C for 1 min, 95 °C for 2 min, and 95 °C for 5 sec, 60 °C for 15 sec, for 40 cycles. The melting curve program temperature was set at 95 °C for 15 sec, 55 °C for 15 sec.

To analyze mRNA expression level in individual 3D spheroid, 2 candidate genes (CDH1, CD44) were measured by using real-time quantitative PCR (q225, Kubo Technology, Beijing, China). After the spheroid generated on the 3D-phd array, we cut the single culture unit off from the array and put it onto a 200 μL clean PCR tube. The print block can firmly lock on the tube due to the design of the SCS (spheroid culture site). The qPCR analysis show that each individual MCF-7 tumor spheroid presented a similar gene expression profile, inferring that retrieval is successfully performed.

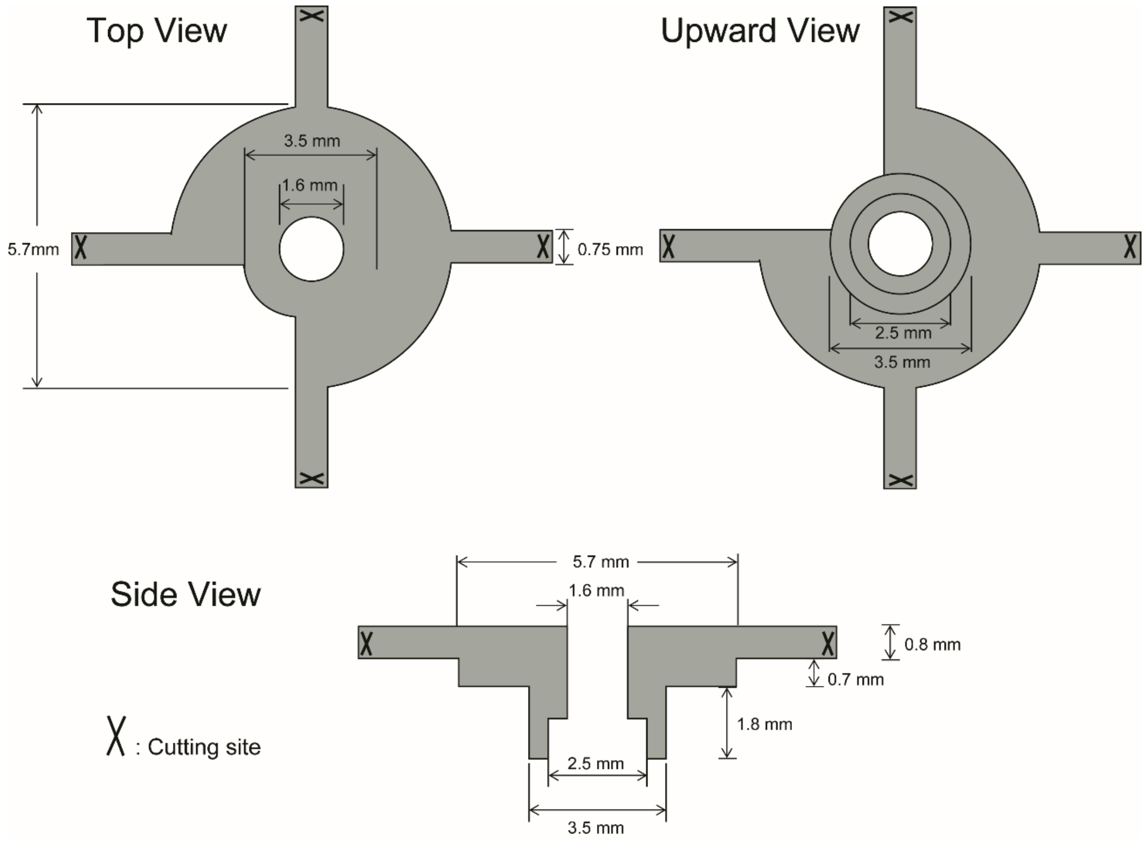

Figure S1. Schematic illustration of the multiview projections of 3D-phd-device (96 well). Series of two-dimensional orthographic view shows the size and architecture of the spheroid culture site (SCS) on 3D printed 96-well array. Each single unit can be cut from the array and locked onto a standard 0.2 mL PCR tube for single spheroid retrieval using a micro-centrifuge as shown in figure S8.

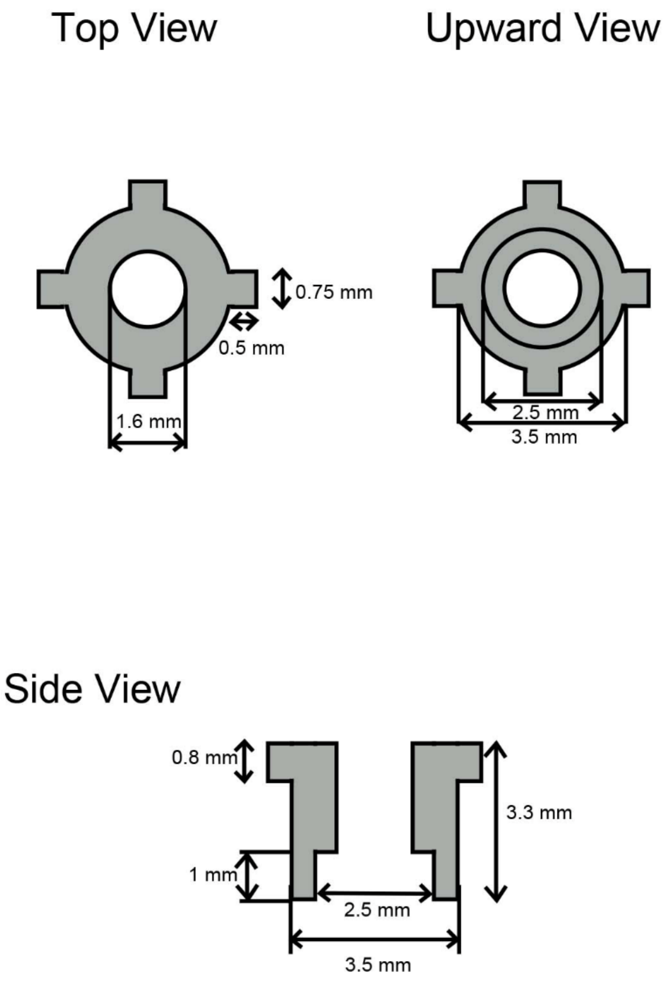

Figure S2. Schematic illustration of the multiview projections of 3D-phd-device (384 well). Series of two-dimensional orthographic view shows the size and architecture of the spheroid culture site (SCS) on 384-well printed array.

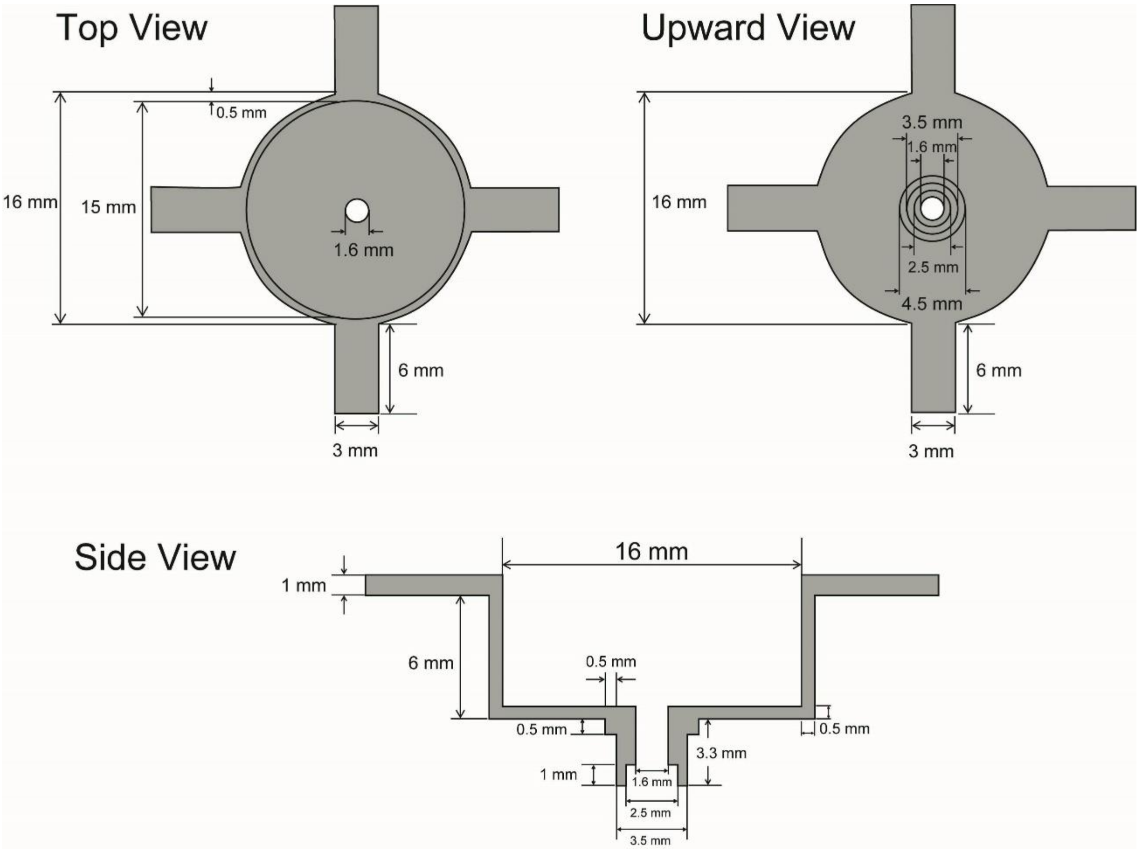

Figure S3. Schematic illustration of the multiview projections of 3D-phd-device (24 well). Series of two-dimensional orthographic view shows the size and architecture of the spheroid culture site (SCS) on our 3D printed 24-well array. This design allows the spheroid located at the central region of the well after dripping-down by using 30 μL medium or ECM gel depending on different assay.

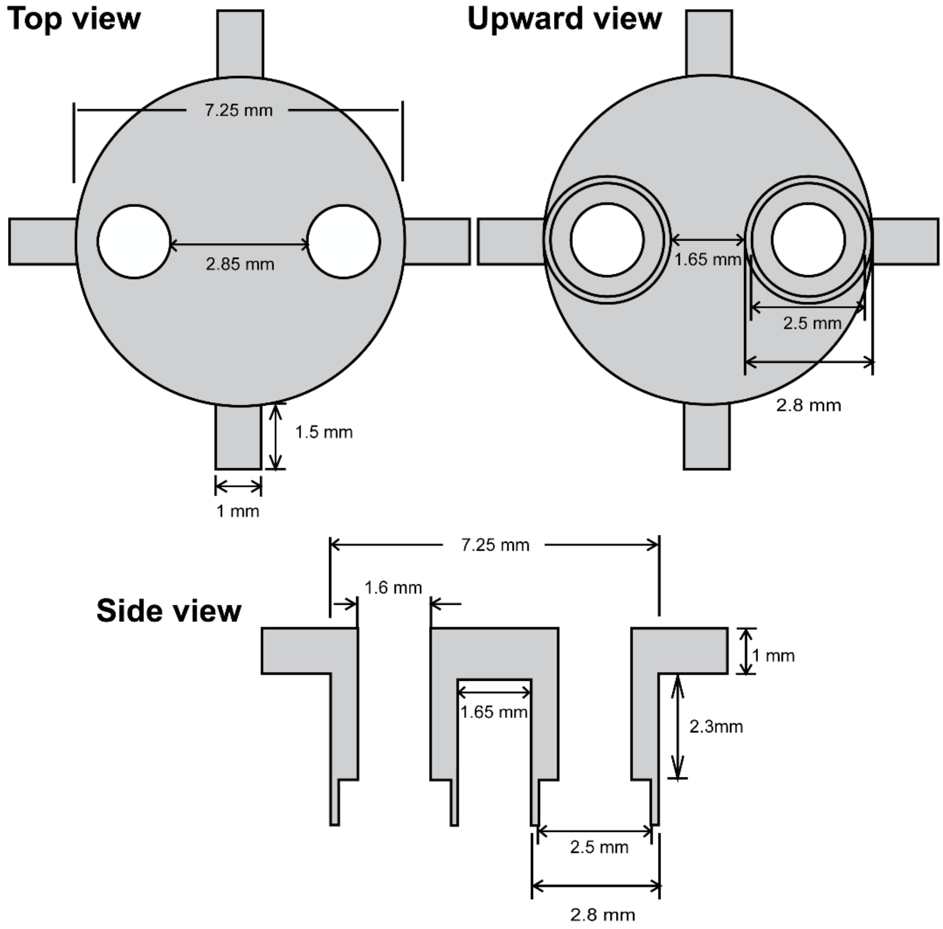

Figure S4. Schematic illustration of the multiview projections of 3D-phd-device (Double nozzles per well). Series of two-dimensional orthographic view shows the size and architecture of the spheroid culture site (SCS) on 3D printed double nozzles per well structure. This design allows two independent hanging drops contains deferent spheroids co-exist in same well after dripping-down. by using medium or ECM gel depending on different assay.

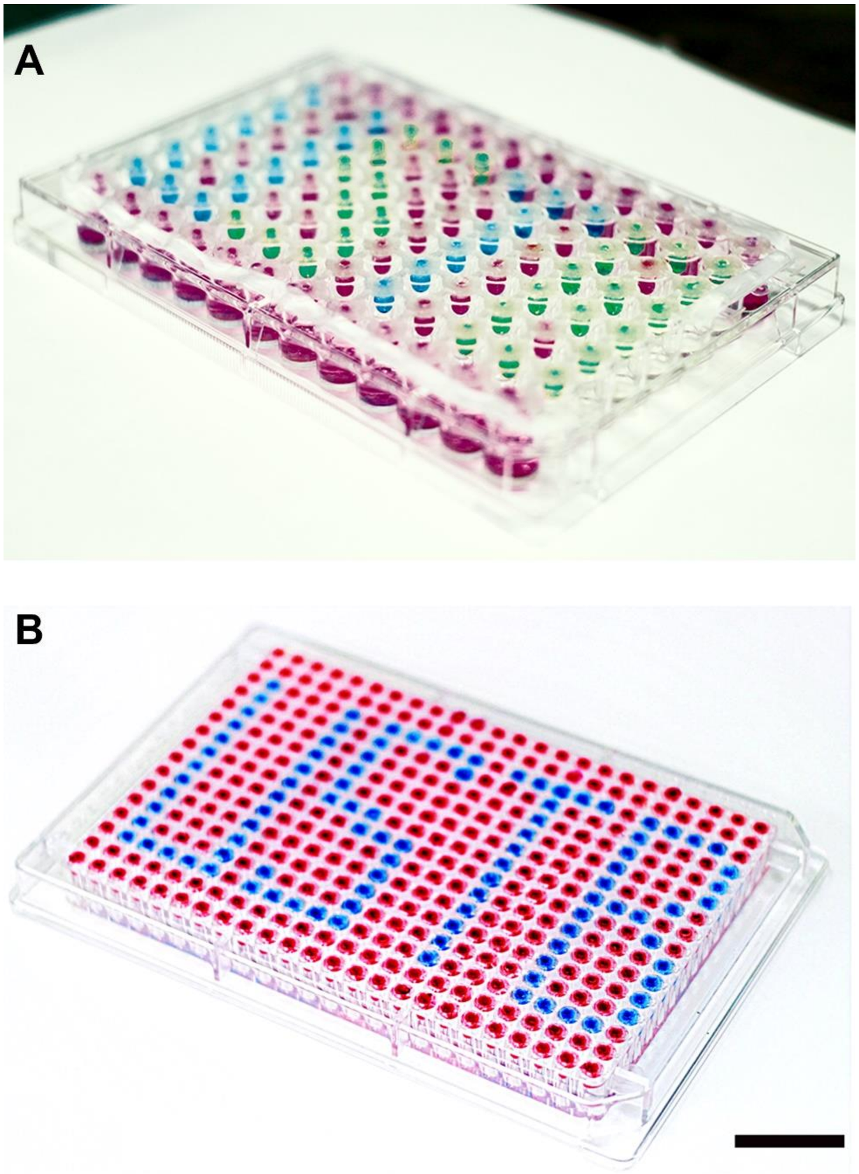

Figure S5. Photograph of colored hanging drops on our 3D-phd device riding on a 96 (A) and 384 plate (B), shows drops alignment for demonstrating initials (USTB) of University of Science and Technology Beijing. Scale bar is 2 cm.

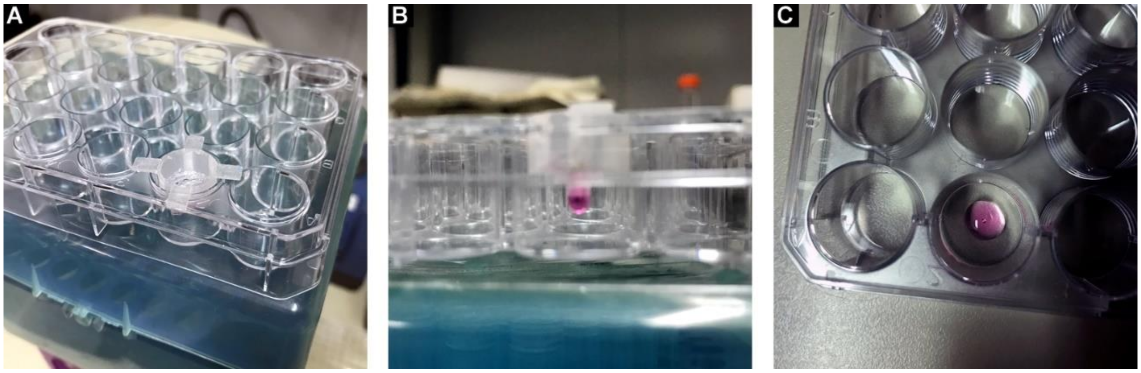

Figure S6. Pictures of 24-well format 3D-phd device. This format allows cell spheroid located at the central region of the downward well after dripping off. The centralized spheroid will facilitate image-based study of cell spheroid dissemination.

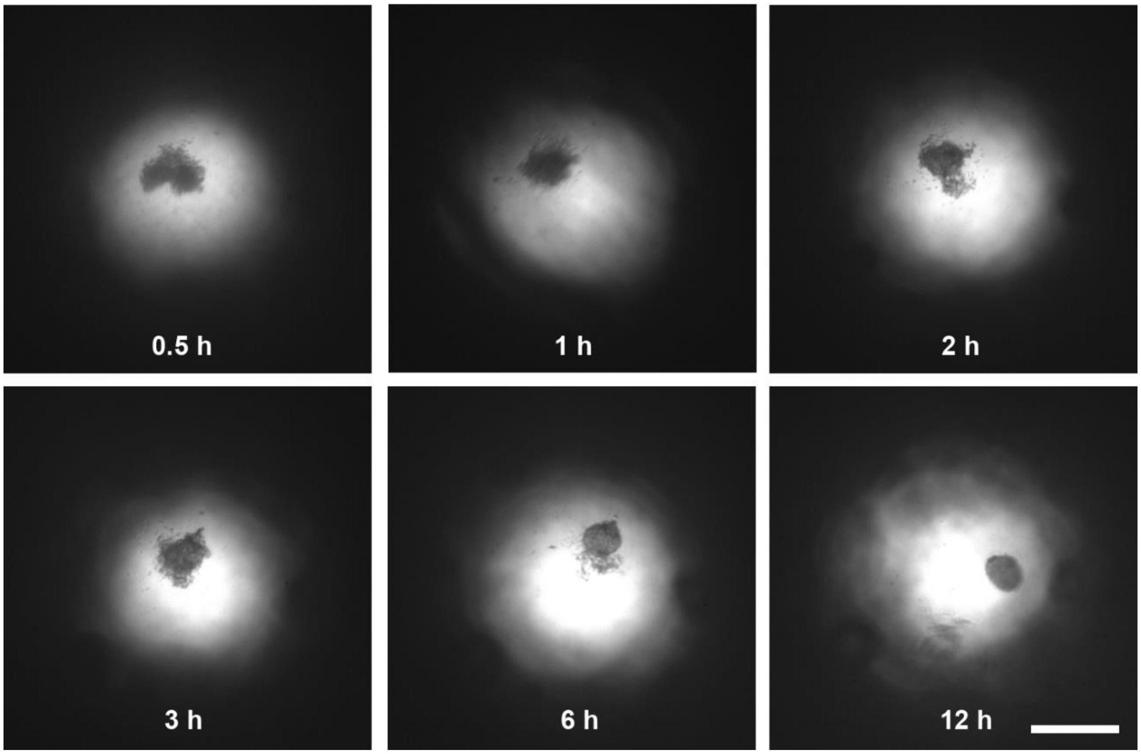

Figure S7. Time-lapse micrographes for demonstrating an individual spheroid formation on a 3D-phd array. The cells are loosely gathered from beginning to 2h. A clear spheroid can be obtained after 12 h seeding on the 3D-phd array (96-well format). Scale bar is 500 μm.

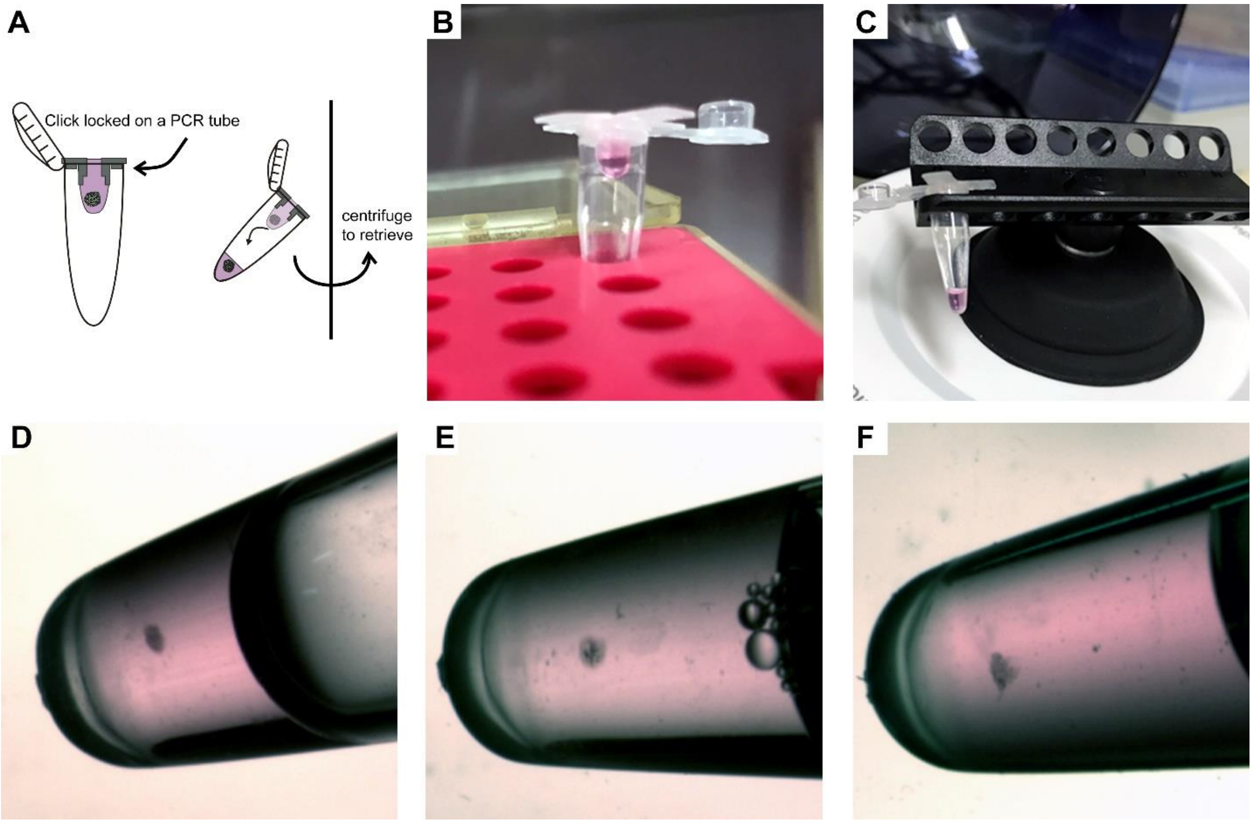

Figure S8. (A) Illustration of the easy retrieval of single spheroid using a 96-well format 3D-phd array. The single unit of 3D-phd can be cut from array and subsequently locked on a 0.2 mL PCR tube for easy retrieval. (B) Picture shows the culture unit can perfectly fit onto a 0.2 mL PCR tube. (C) The single cell spheroid can be obtained by spinning down with a palm-micro-centrifuge. (D)-(F) Micrographes of received individual cell spheroid in the tube in three consecutive recovery using this procedure.

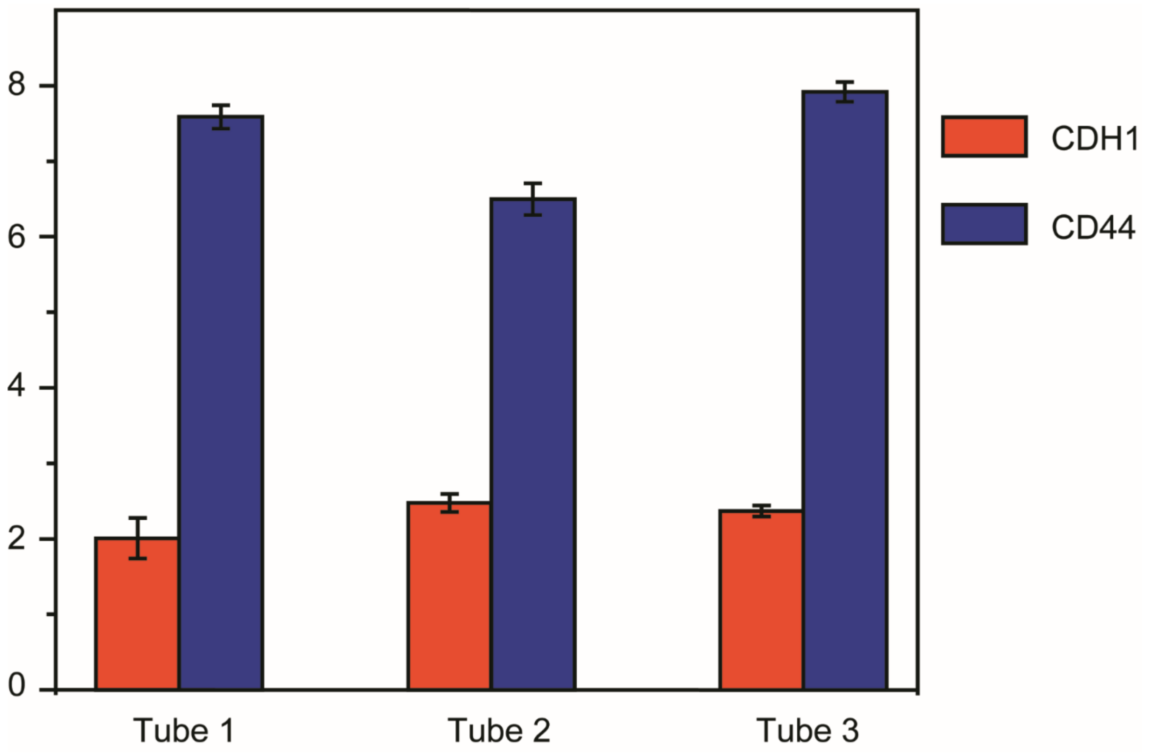

Figure S9. qPCR assay of three consecutive single-spheroids separately retrieved from 3D-phd array, as shown in figure S8. Relative expression (-ΔCt) of 2 genes, CDH1 (E-cadherin) and CD44 (HCAM), have been quantified and GAPDH was served as internal reference gene. Technical replicates in qPCR test, N=6.

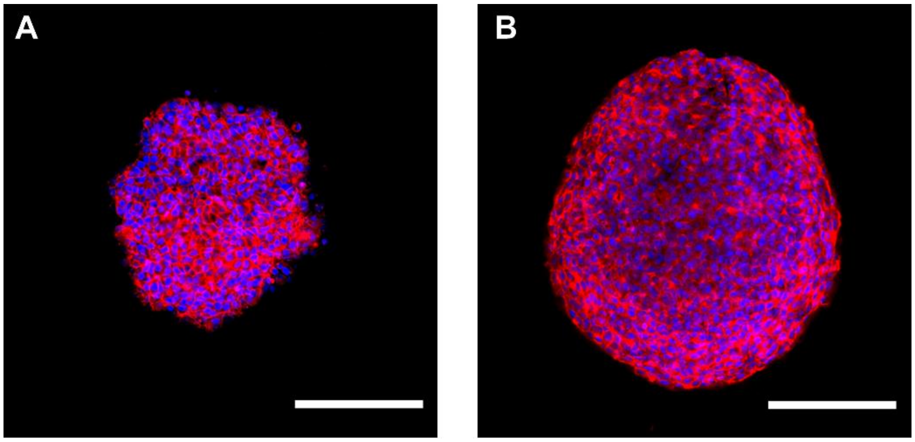

Figure S10. Micrographes of f-actin/nucleic double staining of tumor spheroids formed on 3D-phd array. These images show that the size of spheroid can affect staining evenness. The dye can penetrate the smaller spheroid (~200 μm) whereas difficult to stain the core region of a larger spheroid (~400 μm). Scale bar is 200 μm.

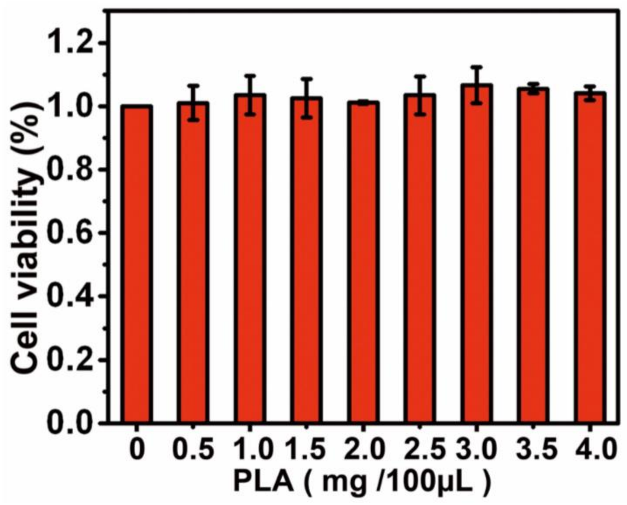

Figure S11. Cytotoxicity test of polylactic acid (PLA) by using MTT assay. No significant effects on cell viability have been detected when adding 0.8 g PLA into 100 μL cell culture medium.

Device format	96-well with ring	96-well without ring	384-well with ring	384-well without ring
Average diameter of cell spheroids	211±12 µm	238±23 µm	227±19 µm	229±26 µm
Spheroids Yield	97±2 %	63±11 %	93±4 %	54±10%

Table S1. Statistical analysis of the spheroids generated on different design of 3D-phd platform. Data was obtained from three independent device for each condition. 1500 cells per seeding spot was used for characterization.

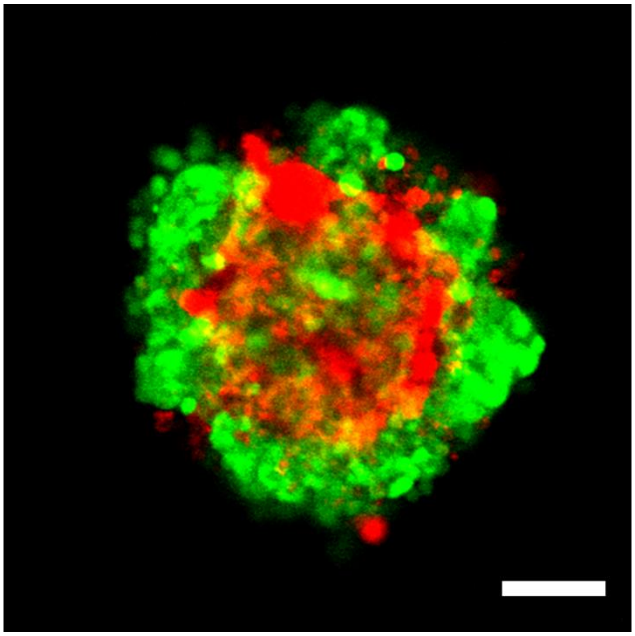

Figure S12. Formation of heterogeneous co-culture spheroids. HCT-116-eGFP cells were culture with HUVEC-tdTomato in hanging-drop device for 2 days. Scale bar is 100 µm.

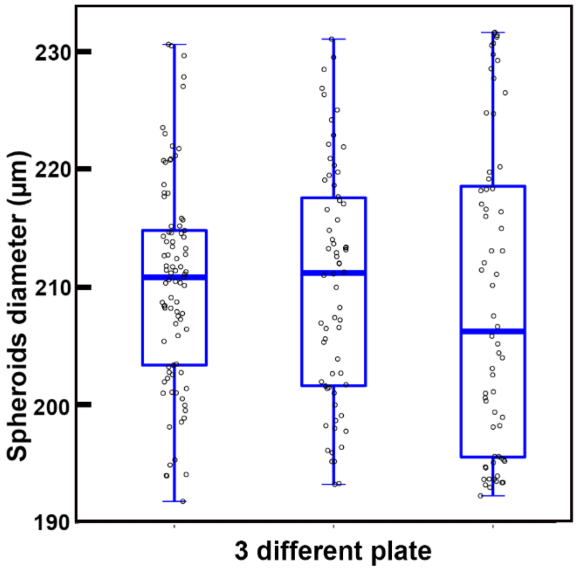

Figure S13. Size distribution analysis of tumor spheroids produced on three different 3D-phd (N=90). 1500 cell/drop was used in each device. Median spheroids diameter was 211 µm, 212 µm, and 205 µm, respectively.

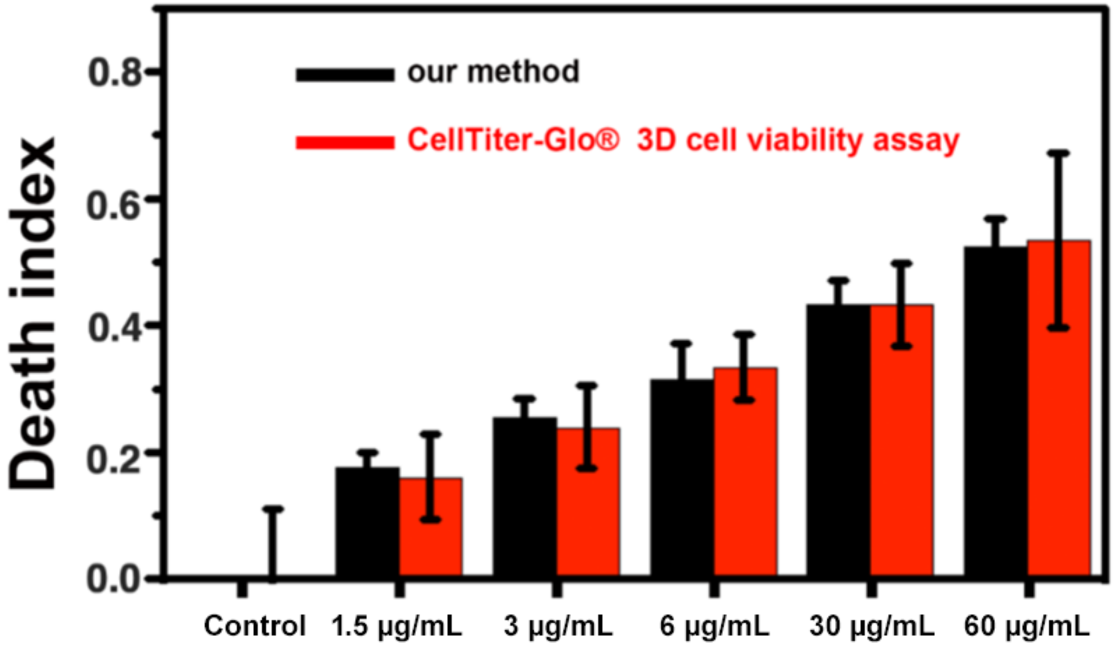

Figure S14. Comparison of cell viability from Cell Titer-Glo 3D reagent (Promega) and double staining. Spheroids were treated with cisplatin at different concentrations for 2 days (n=8).

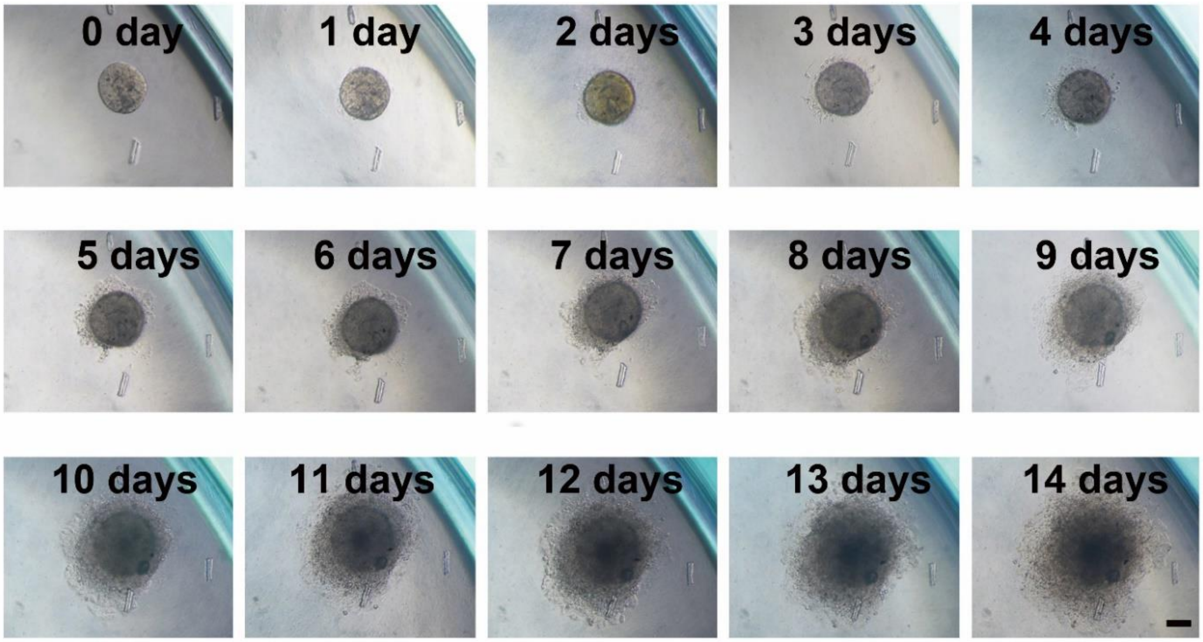

Figure S15. Time-lapse micrographs illustrate the migration of MCF-7 spheroid embedded in collagen gel for 14 days. Scale bar is 100 µm.

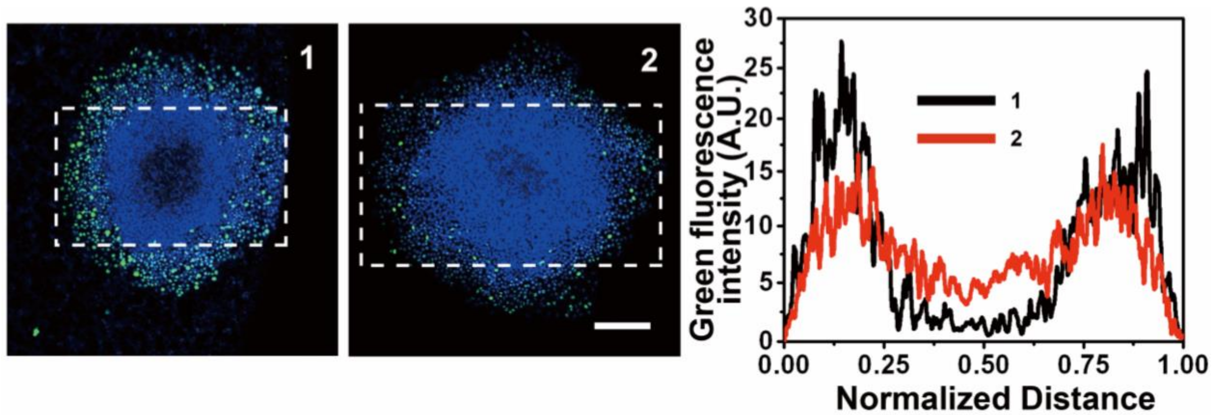

Figure S16. Proliferation analysis of MCF-7 spheroids that have been grafted in collagen gel for 14 days. Ki67 staining distinguished the mitotic cells, and all cell nucleus was stained with Hoechst 33342 (Blue) respectively. The fluorescence intensity was plotted along with the region of interested (ROI), shows that cells in peripheral edge possessed a higher proliferation rate compared with the quiescent core of the tumor spheroids after 14 days in collagen environment. Scale bar is 200 µm.
